# Iodine intake in human nutrition: a systematic literature review

**DOI:** 10.3402/fnr.v56i0.19731

**Published:** 2012-10-09

**Authors:** Ingibjörg Gunnarsdottir, Lisbeth Dahl

**Affiliations:** 1 Unit for Nutrition Research, University of Iceland and Landspitali The National University Hospital of Iceland, Reykjavik, Iceland; 2 National Institute of Nutrition and Seafood Research (NIFES), Oslo, Norway

**Keywords:** iodine, nutritional status, nutritional requirements, nutrition policy

## Abstract

The present literature review is a part of the NNR5 project with the aim of reviewing and updating the scientific basis of the 4th edition of the Nordic Nutrition Recommendations (NNR) issued in 2004. The main objective of the review is to assess the influence of different intakes of iodine at different life stages (infants, children, adolescents, adults, elderly, and during pregnancy and lactation) in order to estimate the requirement for adequate growth, development, and maintenance of health. The literature search resulted in 1,504 abstracts. Out of those, 168 papers were identified as potentially relevant. Full paper selection resulted in 40 papers that were quality assessed (A, B, or C). The grade of evidence was classified as convincing, probable, suggestive, and no conclusion. We found suggestive evidence for improved maternal iodine status and thyroid function by iodine supplementation during pregnancy. Suggestive evidence was found for the relationship between improved thyroid function (used as an indicator of iodine status) during pregnancy and cognitive function in the offspring up to 18 months of age. Moderately to severely iodine-deficient children will probably benefit from iodine supplementation or improved iodine status in order to improve their cognitive function, while only one study showed improved cognitive function following iodine supplementation in children from a mildly iodine-deficient area (no conclusion). No conclusions can be drawn related to other outcomes included in our review. There are no new data supporting changes in dietary reference values for children or adults. The rationale for increasing the dietary reference values for pregnant and lactating women in the NNR5 needs to be discussed in a broader perspective, taking iodine status of pregnant women in the Nordic countries into account.

Iodine is an essential component of the thyroid hormones, thyroxine (T_4_) and triiodothyronine (T_3_), necessary for normal growth, development, and metabolism during pregnancy, infancy and throughout life (1–3). When the physiological requirements for iodine are not met, a series of functional and developmental abnormalities occur, including thyroid function abnormalities. Severe iodine deficiency results in hypothyroidism, endemic goiter and cretinism, endemic mental retardation, decreased fertility, increased prenatal death, and infant mortality (1–4). High iodine intake may also cause disturbances in the thyroid function (1–4).

In the 4th edition of the Nordic Nutrition Recommendation (NNR) (4) issued in 2004, the recommended daily intake (RDI) of iodine was kept unchanged from the 3rd edition (1996). RDI was set to 90 µg/day for children aged 2–5 years, 120 µg/day for children aged 6–9 years, and 150 µg/day for children from 10 years of age, adolescents, and adults. The RDI for iodine presented in NNR 2004 for children, adolescents, and adults is in line with current reference values from different countries and organizations (1, 5). In the 4th edition of NNR, an extra 25 µg/day was recommended during pregnancy (RDI set to 175 µg/day) and extra 50 µg/day during lactation (RDI set to 200 µg/day) to provide sufficient iodine in the breast milk (NNR 2004). These reference values were lower than the reference values of 200 µg/day during pregnancy and 250 µg/day during lactation presented by FAO/WHO in 2005 (1). Furthermore, the WHO/UNICEF/ICCIDD recently increased reference values for pregnant women from 200 to 250 µg/day (6).

The recommended indicator for measuring iodine status is based on the population median urinary iodine concentration (UIC) and iodine intake is regarded as adequate when the UIC is 100–199 µg/L (2, 3). Population iodine sufficiency during pregnancy is defined by median UICs of 150 –249 µg/L (6).

The present literature review is a part of the NNR5 project with the aim of reviewing and updating the scientific basis of the 4th edition of the NNRs (4) issued in 2004 (Nord 2004:13). A number of systematic literature reviews will form the basis for establishment of dietary reference values in the 5th edition of NNR.

## Aims

The overall aim was to review recent scientific data on health effects of iodine status (as an indicator of iodine intake). The specific objectives of the review were to assess the influence of different intakes of iodine at different life stages (infants, children, adolescents, adults, elderly, and during pregnancy and lactation), in order to estimate the requirement for adequate growth, development, and maintenance of health. In collaboration with the NNR5 horizontal group on pregnancy and lactation, we added one specific aim, that is, to assess the scientific evidence and special relevance for the Nordic setting by increasing the RDI of iodine during pregnancy and lactation from what was presented in the 4th edition of NNR.

## Research/key questions

Five research questions were developed:

1. What is the effect of *insufficient iodine intake*, from diet and supplements, on functional or clinical outcomes in different life stages (pregnancy, infancy, childhood, adulthood, and elderly)?
2. What is the effect of *excessive iodine intake*, from diet and supplements, on functional or clinical outcomes in different life stages (pregnancy, infancy, childhood, adulthood, and elderly)?
3. What is the association between *iodine status* (dose response) and clinical and functional or clinical outcomes?
4. What is the effect of *iodine intake from different sources* on iodine status (UIC)?
5. What are the effects of *other nutrients*, such as selenium and iron, on iodine status?

The main functional or clinical outcomes of interest were pregnancy outcome, childhood development (including cognitive function and growth), thyroid function (thyroid hormones, thyroid gland size, hyper- and hypothyroidism), metabolism, health, and weight. See Appendix 1 for search terms. Out of the five research questions, only the studies related to the first three are presented in this review, the reason being lack of data related to research questions four and five.

## Methods

Search terms were defined during spring 2010, in collaboration with Sveinn Olafsson, librarian at Landspitali The National University Hospital of Iceland, Reykjavik, Iceland. The search terms are presented in Appendix 1. The final search was run in September 2010, including all the relevant population groups and clinical outcomes, resulting in 1,516 abstracts. Studies published from January 2000 until September 2010 were included. Abstract screening was conducted in October and November 2010 according to the guide for conducting Systematic Literature Reviews for the 5th edition of the NNRs. Inclusion criteria in the abstract screening process were the following: relevant to iodine nutrition in the Nordic countries, Nordic or English language, ≥50 subjects, representative samples of the population or specific sub-samples of the population, preferably using UIC (spot samples or 24-h collections) as indicator of iodine status. Other potential indicators of iodine status and thyroid function, such as thyroid volume (TV), thyroid-stimulating hormone (TSH), T3 and T4, were also included. Most cross-sectional studies, only describing iodine status without clinical outcomes of interest for this review, were excluded at this point. Exceptions were studies conducted in one of the Nordic countries or studies with clinical outcomes of interest that might not be covered by data from cohort studies or intervention trials.

The overall aim of the present work was to review and update the scientific basis of the NNRs (NNR 4th edition), issued in 2004 (Nord 2004:13). As a systematic review was not used as basis for the NNR 2004, we decided to order some review papers along with original papers. The reason for this decision was also related to the special aim of the current review to assess the scientific basis for recently increased reference values from WHO/UNICEF/ICCIDD for pregnant women (6), and the relevance for the Nordic setting. All together 276 full papers were ordered, of which 108 papers were immediately excluded and not included in the full paper selection (86 overviews, 19 editorials, commentary, prize lectures, opinions or letters to the editors, and 3 publications that had been withdrawn), leaving 168 publications. Full paper screening was conducted in February 2011, where 128 papers were excluded, leaving 40 papers selected for quality assessment. Reasons for exclusion are provided in Appendix 2. The selected papers were grouped according to clinical outcomes and different age stages into the following categories: pregnancy and lactation, including endpoints such as birth outcome, development, and health of the offspring (*n*=16); children, including endpoints such as cognitive function and development (*n*=9); excessive iodine intake (*n*=4); and adults (*n*=2). Studies from the Nordic countries (*n*=13) were assessed separately in order to get an overview of iodine nutrition in the Nordic countries. Many of the Nordic studies only included descriptive information, while others were included in the relevant categories (according to clinical endpoints presented in each paper) at a later stage (*n*=4, all in the pregnancy and lactation category).

To evaluate the quality of the selected articles (*n*=40), we used the Quality Assessment Tool (QAT) received from the NNR5 secretary. The QAT included questions about study design, recruitment, compliance, dietary assessment, confounders, statistics, outcomes, and so on. The summary of findings from studies graded as A or B according to QAT are presented in summary Tables 1–6. Detailed information is provided in evidence tables (Appendix 3–7). Main results of the papers graded C are given in the text, but those studies are not used in the final grading of evidence. The grade of evidence was classified as convincing, probable, suggestive, and no conclusion, in line with criteria introduced in the Systematic literature review (SLR) guide for the 5th edition of NNR.

**Table 1:** Summary table. Iodine status and iodine supplementation in pregnancy; pregnancy outcome and thyroid function in the mother and offspring

Author, year, (reference number.)	Population	No. of participants	Intervention/exposure	Outcome variable	Effect	Study quality	Comments
Antonangeli et al. 2002 (7)	Pregnant women	*n*=67	Supplementation of 200 µg/day vs. 50 µg iodine	UIC/TV	Six months after delivery UIC was 230 µg/g creatinine in group A and 128 µg/g creatinine in group B. NS difference in TV.	B	Observed difference in UIC after randomization, but no statistical test reported on if this difference is statistically significant.
Nøhr & Laurberg, 2000 (9)	Pregnant women/Infants	*n*=144	150 µg iodine supplement (+I) or no iodine (no I).	TSH	Mothers in the +I group had lower TSH (mU/L), than the no I group. The +I group of neonates had higher TSH than the no I group.	B	The study suggests that iodine supplementation of the mother will, in general, not improve fetal thyroid function in areas such as Denmark with mild iodine deficiency. A slightly inhibitory effect may be expected, which is probably not of clinical significance.
Nøhr et al., 2000 (8)	Pregnant women with thyroid peroxidase antibodies (TPO-Ab)	*n*=66	150 µg iodine supplement or no iodine supplementation.	Postpartum thyroid dysfunction (PPTD)	TPO-AB level at screening was a good predictor of the PPTD risk. No statistical significant difference in the frequency of PPTD in the three groups, with no significant increase in the prevalence, severity, or duration of PPTD when 150 mg iodine was given to TPO.	A	Unlikely that supplementation of 150 µg/day will have adverse effects in TPO-Ab women living in an area with mild to moderate ID.

SGA=short for gestational age, TV=thyroid volume, TSH=thyroid-stimulating hormone.

**Table 2:** Summary table. Thyroid function in pregnancy and cognitive function in infancy and childhood

Author, year, (reference number)	Population	No. of participants	Intervention/Exposure	Outcome variable	Effect	Study quality	Comments
Choudhury & Gorman, 2003 (15)	Children, 7 months and 13 months	*n*=135	Prenatal iodine deficiency (cord blood TSH concentration); control group <5 mU/L, group 2 10.0–19.9 mU/L, group 3 20.0–29.9 mU/L and group 4 ≥30 mU/L.	Mental development index (MDI)	Infants in groups 3 and 4 had lower novelty preference (suggesting less efficient information processing) than infants in the non-elevated and the mildly elevated groups at 7 months. The three elevated TSH groups (2, 3, and 4) had significantly lower MDI scores than the non-elevated control group at 13 months.	B	No information on iodine nutrition (neither UIC nor iodine intake).
Oken et al., 2009 (16)	Children, 6 months and 3 year	*n*=500	Newborn T_4_ (thyroxine) levels and maternal thyroid function (plasma TSH, total T_4_ and TPO antibody levels)	Visual recognition memory (VRM) paradigm at 6 months. Cognition assessed at 3 year.	Higher newborn T_4_ was associated with slightly lower scores on the VRM test at 6 months. Newborn T_4_ levels were not associated with cognitive tests at 3 year. No evidence that impaired maternal thyroid function was associated with lower child cognitive test scores.	A	No information on iodine nutrition (neither UIC nor iodine intake).

KI=potassium iodide; TSH=thyroid-stimulating hormone; UIC=Urine iodine concentration.

**Table 3:** Summary table. Iodine supplementation or improved iodine status in childhood and cognitive function

Author, year, (reference number)	Population	No. of participants	Intervention/exposure	Outcome variable	Effect	Study quality	Comments
Gordon et al., 2009 (23)	Children, 10–13 years	I group *n*=84 and placebo *n*=82.	150 µg I tablet vs. placebo	Cognitive performance	Cognitive performance improved in the I group (2 out of 4 subtests)	B	Relevant in a Nordic perspective since the study is among children in mildly iodine-deficient area.
Zimmermann et al., 2006 (24)	Children 10–12 years	I group *n*=159 and placebo *n*=151.	400 mg iodine as oral iodized oil vs. placebo.	Cognitive and motor performance	Cognitive and motor performance improved in the I group (4 out of 7 subtests)	B	Study from an iodine-deficient area in Albania. Might not be relevant for the Nordic countries.
Van den Briel et al., 2000 (25)	Children 6–12 years	Improved group (*n*=128), unchanged group (*n*=68).	Iodine status changed from severe iodine deficiency to moderate, from severe to normal-mild, or from moderate to normal-mild.	Mental and psychomotor performance	Greater increase in performance on the combination associated with improved iodine status	B	Study includes schoolchildren in Benin and reflects not Nordic countries

I=iodine.

**Table 4:** Summary table. Iodine status and health outcomes in adults and elderly

Author, year, (reference number)	Population	No. of participants	Intervention/Exposure	Outcome variable	Effect	Study quality	Comments
Ayturk et al., 2009 (32)	Newly diagnosed patients with metabolic syndrome (18–74 years) and controls living in a mild-to moderate iodine deficiency area	*n*=539; *n*=278 in the metabolic syndrome group (33.1% male) and *n*=261 in the control group (30.7% male)		Thyroid volume	TSH was significantly correlated with the presence of metabolic syndrome. Insulin resistance, waist circumference and triglycerides independent predictors of thyroid volume.	B	No information on iodine nutrition (neither urine iodine nor iodine intake).
Hoption Cann et al., 2007 (33)	Males 25–74 years	*n*=4,234 (*n*=197 cases)	Tertiles of iodine/creatinine categories (<201 µg/g *n*=1,452, 201–345 µg/g *n*=1,554, >345 µg/g *n*=1,228, referred to as low, moderate and high levels	Prostate cancer incidence	Risks of prostate cancer between tertiles of Iodine/creatinine categories NS after adjustments for potential confounding factors. History of thyroid disease was associated with greater than twofold increased risk.	B	The role of iodine remains speculative. A role of thyroid disease and/or factors contributing to thyroid disease as a risk factor for prostate carcinogenesis warrants additional investigation.

**Table 5:** Summary table. Excessive intake of iodine

Author, year, (reference number)	Population	No. of participants	Intervention/exposure	Outcome variable	Effect	Study quality	Comments
Zimmermann et al., 2005 (34)	Children 6–12 years	*n*=3,319	UIC <300 µg/L, UIC 300–500 µg/L, UIC >500 µg/L	Thyroid volume (TV)	UIC of 300–500 µg/L not associated with increased TV. TV started to increase at a UIC ≈ 500 µg/L.	B	The authors don‘t rule out adverse effects of UIC in the range of 300–500 µg/day not detected in this study

UIC=urinary iodine concentration.

**Table 6:** Summary table. Mandatory salt iodization in Denmark

Author, year, (reference number)	Population	No. of participants	Intervention/exposure	Outcome variable	Effect	Study quality	Comments
Pedersen et al., 2002 (38)	Adults 18–65 years	*n*=310,124 in the Aalborg area and *n*=225,707 in the Copenhagen area	Mandatory salt iodization (13 µg/g)	Incidence rates of hyper- and hypothyroidism	Iodine supplementation may increase the incidence of overt hypothyroidism even if the population is moderately iodine deficient.	B	The optimal level of iodine intake to prevent thyroid disease may be a relatively narrow range around 150 µg/day.
Rasmussen et al., 2009 (39)	Adults 18–65 years	*n*=4,649 and *n*=3,570	Mandatory salt iodization (13 µg/g)	UIC and FFQ	Iodine excretion (µg/L and µg/24 h) increased significantly in all age and sex groups.	B	Iodine intake is at recommended level, however in the youngest age groups in both cities and in women aged 40–45 year living in Aalborg, the iodine intake is below the recommendation. No groups with excessive iodine intake were identified.
Vejbjerg et al., 2007 (40)	Adults 18–65 years	*n*=4,649 and *n*=3,570	Mandatory salt iodization (13 µg/g)	Thyroid volume	Lower thyroid volume in all age groups	B	The decline in thyroid volume was largest in the regions with former moderate iodine deficiency.
Vejbjerg et al., 2008 (45)	Adults 18–65 years	*n*=4,649 and *n*=3,570	Mandatory salt iodization (13 µg/g)	Thyroid volume and TSH in relation to smoking.	Smokers have lager thyroid volume than non-smokers; however, the difference in thyroid volume was reduced after iodization. The effect of smoking on TSH and free T_4_ was unchanged after iodization.	B	The effect of smoking on thyroid volume seems to be dependent on iodine intake.
Vejbjerg et al., 2009 (41)	Adults 18–65 year	*n*=4,649 and *n*=3,570	Mandatory salt iodization (13 µg/g)	TSH and prevalence of thyroid dysfunction	Higher TSH level after iodization in both regions and across age groups. Lower prevalence of mild hyperthyroidism and increased prevalence of hypothyroidism related to a higher iodine intake.	B	Increased iodine intake after mandatory iodization change the pattern of thyroid dysfunction in the population.

UIC=urinary iodine concentration, FFQ=food frequency questionnaire, TSH=thyroid-stimulating hormone.

## Results

### Pregnancy and lactation

#### Iodine status and thyroid function

Studies relating iodine status during pregnancy to maternal and/or neonatal thyroid function are presented in Table 1 (details are provided in Appendix 3). An Italian trial (7) assessed iodine status and thyroid function in women after supplementation of 200 µg iodine or 50 µg iodine per day during pregnancy and up to 6 months after delivery. Improved iodine status was observed in both groups, but no difference in thyroid function was found between groups. The most relevant studies in the Nordic perspective are those from Denmark (8, 9). The study by Nøhr and Laurberg (9) included healthy pregnant women with no previous history of thyroid disease, comparing maternal and neonatal thyroid function between mothers receiving 150 µg iodine as a supplement during pregnancy to those not receiving any supplements. Although small difference in thyroid function was seen between groups, the study suggests that iodine supplementation of the mother will, in general, not improve fetal thyroid function in areas such as Denmark with mild iodine deficiency. A randomized controlled trial was conducted by the same research group among women with thyroid peroxidase antibodies (TPO-Ab), showing that it is unlikely that supplementation of 150 µg/day will have adverse effects in TPO-Ab women living in an area with mild-to-moderate ID (8).

Iodine nutrition of pregnant women from Norway (*n*=119) was studied by Brantsæter (C-study) and colleagues (10). Women using dietary supplements had median iodine intake of 215 µg/day (range 106–526) compared with 122 µg/day (range 25–340) among non-supplement users. The median UIC was also significantly higher in iodine supplement users (190 µg/24 h for FFQ and 220 for FD) than in non-supplement users (110 µg/24 h) (10).

#### Pregnancy complications and pregnancy outcomes

All studies in this category were evaluated as low-quality studies (C) due to high drop-out rate, or other methodological issues (data not shown). Higher birth weight of infants whose mothers had UIC 50–99 µg/L compared with those with UIC < 50 µg/L was reported in a cohort study from Spain (11). Three more studies assessed the association between iodine status and reproductive failure (12) or pregnancy complications (13–14).

#### Cognitive function

Table 2 (details are provided in Appendix 4) describes studies relating prenatal indicators of iodine status to cognitive function in the offspring. In the study by Choudhury and Gorman (15), Chinese infants were stratified into iodine deficiency groups (ID) by cord blood TSH concentration. Lower mental developmental index (MDI) was observed in the group with highest cord blood TSH. The third study in Table 2 describes results from Project Viva (16) where associations between maternal as well as newborn thyroid function and cognitive function were assessed. Higher level of T_4_ in newborns was associated with slightly lower scores on the visual recognition memory test at 6 months. However, no association was observed between maternal or newborn thyroid function and cognitive function at 3 years. It should be noted that low number of women had abnormal thyroid function in the study. Other studies in this category were quality graded as C-studies, as the statistical analysis was questioned or potential confounding factors not adjusted for (data not shown). The Berbel study (17) was a non-randomized intervention study where iodine supplementation (200 µg KI/day) was initiated at 4–6 weeks or 12–14 weeks of pregnancy or after delivery. The study suggests that delay in maternal iodine supplementation increases the risk of neurocognitive developmental delay of their offspring. Only 11–12% of the total study population was included in the analysis as the authors established extensive exclusion criteria in order to obtain comparably homogenous groups of children. In a non-randomized intervention study by Velasco and colleagues from 2009, pregnant women were provided with 300 µg iodine in the intervention group, while a control group received no supplementation. Psychomotor development index (PDI, which is one of three scales of the Bayley Scales of Infant Development used in the study) was significantly higher in children of mothers in the intervention group than the control group (18). However, lactation was found to be a confounding factor explaining the variance in the PDI. Other possible confounding variables were not controlled for and the results should therefore be considered as preliminary. In a study from China, cognitive function was assessed in children (5- go 7-year-old follow up) whose mothers initiated iodine supplementation during different stages of pregnancy (early: 1st, 2nd or late: 3rd trimester) and in a control group of children receiving iodine supplementation from 2 years of age (19). The main results point towards the suggestion that children would benefit from their mothers iodine supplementation during pregnancy in the particular population studied.

#### Lactation

The literature search did not result in many papers related to lactation, and only three papers in this area were selected for quality assessment. A Danish study from 2004 (B study according to quality assessment), that was already included in the NNR 4th edition (4, 20), showed that the level of iodine in the breast milk of smokers was 26.0 µg/L (23.2–29.1 µg/L) and in non-smokers 53.8 µg/L (49.4–58.5 µg/L), *p <* 0.001. Significant differences were also found in the infants, as the urinary iodine in infants with smoking mothers was 33.3 µg/L (29.9–37.2) versus 50.4 µg/L (46.0–55.1 µg/L) in non-smokers. Although the main message to breastfeeding mothers would be not to smoke, this study highlights the importance of obtaining enough iodine from the diet or through supplementation.

Several methodological issues (such as low participation rate and lack of adjustments for potential confounders) where observed during quality assessment of the other two studies in this category (21–22). UIC was higher in formula-fed infants than breastfed in a study from New Zealand, although no information was provided on the iodine status of the lactating mothers (21). In an Australian study, a correlation between iodine status of the mothers and iodine content of breast milk was found (22).

### Children

#### Cognitive function

Results of three studies are presented in Table 3 (details are provided in Appendix 5) (23–25), all suggesting improved cognitive function in 6- to 13-year-old children related to iodine supplementation or improved iodine status. The results from the Gordon study, performed in New Zealand, might be relevant in the Nordic setting since the study includes children from a mildly iodine-deficient area (UIC 63 µg/L at baseline). The study suggests that mildly iodine-deficient children might benefit from iodine supplementation of 150 µg/day, in order to attain their full intellectual potential. However, the two other studies might not be relevant in the Nordic perspective, including children from iodine-deficient area of Albania and North Benin. A cross-sectional study from Spain points in the same direction (26), where an intelligence quotient below the 25th percentile was significantly related to UI below 100 µg/L (OR 1.4, *p*=0.02), adjusted for potential confounding factors (data neither shown in Table 3 nor included in grading of evidence).

#### Other outcomes

Only cross-sectional studies were retrieved studying the relationship between iodine status or iodine supplementation and outcomes such as hearing (27), body composition (28, 29), growth, and insulin-like-growth factor-I (30). References to these studies are only included in this review for informational purpose as cohort studies or intervention studies were lacking (data not shown). In an intervention study by Zimmerman (graded as B study) iodine-deficient children (UI at baseline 46 µg/L) were supplemented with iodized oil or iodized salt for 5–6 months. A significant increase was observed in UI in the iodine group (UI 158 µg/L at endpoint), while total and LDL-cholesterol concentration as well as C-peptide decreased (data not shown) (31).

### Adults and elderly

The literature search did not result in many studies, including adults and elderly in relation to iodine. Only two publications were selected for quality assessment in this category, both graded as B studies (Table 4, details are provided in Appendix 6). Subjects with the metabolic syndrome were found to have increased TV and nodule prevalence, and insulin resistance was suggested as an independent risk factor for nodule formation in an iodine-deficient environment (32). However, no information was provided on iodine nutrition (neither urine iodine nor iodine intake), making the study less relevant for the purpose of NNR. Prostate cancer incidence according to UIC concentration (7- to 21-year follow-up) was assessed in the First National Health and Nutrition Examination Survey Epidemiological Follow-up Study (NHEFS) (33). After adjustments for potential confounding factors, the association found turned out to be non-significant. However, reported history of thyroid disease was associated with greater than two-fold increased risk of prostate cancer.

### Excessive intake

Four studies related to excessive iodine intake were a subject to quality assessment by the group. In children, UIC≥500 µg/L was found to be associated with increasing Tvol in 6- to 12-year-old children, while UIC 300–500 µg/L was not (34) (Table 5, details are provided in Appendix 7). Results of other selected papers in this category should be interpreted with caution due to lack of information, especially related to adjustments for potential confounding factors (35–37) (data not shown). A prospective community-based survey among 13-year-old Chinese children, examined again 5 years later, found no difference in occurrence of autoimmune hyperthyroidism between communities with median UIC of 88, 214, and 634 µg/L (35). A case–control study (36) showing small but significant difference in UIC between women with autoimmune subclinical hypothyroidism and the matched controls (327±113 vs. 274±99 µg/L, *p <* 0.01), and a Chinese cohort study by Guan et al. (37) suggested that post-partum thyroiditis (PPT) in pregnant women is triggered by high (defined as UIC > 300 µg/L) iodine intake.

### Iodine nutrition in the Nordic countries

The majority of the studies in the area of iodine nutrition from the Nordic countries are from Denmark. In total, 13 studies from Nordic countries were selected for quality assessment. Results of four of them have already been presented in the section on pregnancy and lactation (8–10, 20). Main results of the studies from the Nordic countries are presented in Tables 6 and 7.

**Table 7:** Iodine status: Studies from the Nordic countries published in year 2000–2010

Country, author, year (reference number)	Location, year of study	Method	Number of subjects and gender	Age (years)	Iodine excretion and thyroid function	Iodine intake
Denmark, Rasmussen et al. 2008 (39)	Aalborg and Copenhagen 1997–1998 and 2004–2005.	FFQ, spot urine and estimated 24 h I excretion	4,649 subjects in 1997–1998 (before fortification) and 3,570 comparable subjects in 2004–2005 (after fortification).	18–65	Median I excretion (25th, 75th): From non-fortified food intake: 1997–8: 61 µg/L (34, 101) or 94 µg I/24 h (60, 159). 2004–5: 101 (57, 151) or 145 µg I/24 h (100, 146). From non-supplement users: 1997–8: 78 µg I/24 h (52, 116). 2004–5: 128 µg/L (92, 183). From supplement users: 1997–8: 157 µg I/24 h (92, 267). 2004–5: 222 µg I/24 h (145– 346).	Median I intake (25th, 75th) from non-fortified food: 1997–8: 109 µg/day (79, 149). 2004–5: 110 µg/day (82, 146). Milk was the strongest determinant of I intake.
Iceland, Gunnarsdottir et al. 2010 (48)	Reykjavik, June 2007–2008	FFQ as personal interview, spot urine and blood.	112 adolescent girls	Born 1987–1992	Median 200 µg/L (20th was 90 and 80th was 320). Average TSH of 2.0±1.2 mU/L.	Average dietary I intake was 170 µg/day. 10% had I intake below 70 µg and none was above 600 µg. (Milk and dairy products provided 43%, fish 24% and supplements 9% of the total dietary I).
Norway, Brantsæter et al. 2007 (10)	Pregnant women in MoBa study at Bærum Hospital.	FFQ, 4-day weighed food record and 24-h urine sample.	119 women	23–44, mean age of 31.	Supplement users FFQ: UI of 180±75 and median 190 µg/24 h. Supplement users food diary: UI 220±110 and median 220 µg/24 h. Non-supplement users: UI 140±90, median 110 µg/24 h.	Supplement users: FFQ I intake of 211±86, range 106–526 µg/day. Food diary I intake of 214±64, range 103–355 µg/day, Non-supplement users: FFQ I intake of 138±38, range 25–340 µg/day. Food diary I intake of 117±42 and range 42–222 µg/day.
Norway, Dahl et al. 2003 (50)	Tromsø, 1999 and Bergen, 2001	Casual urine TSH and free T_4_	32 men and 28 women in Tromsø. 9 men and 35 women in Bergen.	23 – 64 in Tromsø and 21– 49 in Bergen.	Tromsø: 132 (38–572) µg I/L in men, 112 (57–314) µg I/L in women TSH 1.4 mIU/L in men, 1.8 mIU/L in women Free T_4_ 15 pmol/L in men and women. Bergen: 106 (25–182) µg I/L or 140 (33–235) µg I/24 h in men, 82 (8–348) µg I/L or 79 (16–316) µg I/24 h in women, TSH 1.3 (0.5–4.2) mIU/l in men and women, free T_4_ 12 (10–16) pmol/l in male and women	Tromsø: Men 187 and women 114 µg I/day. Bergen: Men 147 and women 75 µg I/day.
Sweden, Anderson et al. 2009 (46)	Representative national samples collected between Oct 2006 and May 2007.	Spot urine	857 school aged children. 445 boys and 412 girls.	6–12	Median UIC was 125 µg/L, range 11–757. 36% had <100 µg/L, 3% had >300 µg/L.	NA
Sweden, Milakovic et al. 2004 (47)	Semi-rural community (Mölnlycke)	Spot urine Thyroid volume	Group 1: 38 boys and 23 girls. Group 2: 12 boys and 38 girls. Group 3: 57 adults	Group 1: 7–9. Group 2: 15–17. Group 3: 60–65.	Median urinary iodine concentration was in group 1: 194 µg/L, group 2: 246 µg/L and group 3: 190 µg/L. Median (range) of thyroid volume was in group 1: 4.7 mL (2.9–17), in group 2: 11.5 mL (5.6–32) and in group 3: 14.3 mL (3.1–33).	NA

FFQ=food frequency questionnaire, I=iodine, TSH=thyroid-stimulating hormone, UIC=urinary iodine concentration, T4=thyroxine.

#### The effect of iodization of salt on iodine status in Denmark

The Danish Investigation of Iodine Intake and Thyroid Disease (DanThyr) is the official clinical monitoring of the Danish iodine supplementation program, which prospectively measure the incidence rates of hyper- and hypothyroidism in the cities of Aalborg and Copenhagen. In the first examination in 1997–98, the Aalborg area was found to be in the range of moderate iodine deficiency, whereas the area around Copenhagen had mild iodine deficiency (38). The difference in iodine intake in these two areas can mainly be explained by the difference in iodine content in drinking water (5 µg/L in Aalborg and 18 µg/L in Copenhagen) (39). In 2000, it became mandatory to fortify all salts used in bread and household with iodine at a level of 13 µg/g. In 2004–2005, the urinary iodine excretion had increased significantly in all age groups compared with before mandatory iodine fortification in both areas. For instance, the median-estimated 24-h urinary iodine excretion in both areas was 78 µg/day before iodization and 140 µg/day after iodization among non-supplement users. The corresponding median UIC in both areas increased from 61 µg/L in 1997–1999 to 101 µg/L in 2004–2005 (39). However, the iodine intake in the youngest age groups in both cities and in women aged 40–45 years in the Aalborg area was still below the recommendation after the mandatory iodization of salt (39). Milk, water, and salt intake were determinants of iodine intake in 2004–2005, whereas bread and fish intake were not related with iodine intake (39).

#### Associations between iodine status and thyroid function

The studies from Denmark based on the DanThyr programme shows marked differences in pattern of thyroid dysfunction with different iodine intakes (40, 41) and the optimal level of iodine intake to prevent thyroid disease may be a relatively narrow range around the recommended daily iodine intake of 150 µg (42). In general, mild and moderate iodine deficiency is associated with more hyperthyroidism and less hypothyroidism than high iodine intake (42). In 1997–1998, the incidence rate of hyperthyroidism was higher in the Aalborg area with moderate iodine deficiency (with UI of 45 µg/L) compared with the Copenhagen area with higher iodine intake (mild iodine deficiency) (with UIC of 61 µg/L) (38). Further, hyper- and hypothyroidism were more common in females than in males in both areas, and the incidence rates of both hyper- and hypothyroidism increased with age. In the Copenhagen area, a higher incidence rate of hypothyroidism was found compared with the Aalborg area.

Even the small differences in UIC from mild (61 µg/L) and moderate (45 µg/L) iodine deficiency areas in Denmark showed marked differences in the prevalence of goiter with 9.8% goiter in the mild iodine deficiency area (Copenhagen) and 14.6% goiter in the moderate iodine deficiency area (Aalborg) (43, 44).

A lower TV was seen in all age groups independent of sex after iodization and the decline was largest in the Aalborg area with former moderate iodine deficiency (40). The level of TSH was also found to increase from 1.30 mUI/L to 1.51 mUI/L in both regions and across age groups after the introduction of iodization of salt (41). The increase was expected as populations with iodine sufficiency in general have a higher level of TSH than populations with iodine deficiency (41). The effect of smoking on hormonal levels of TSH and free T_4_ were unchanged after the iodization, however, increased iodine intake had an effect on the TV of smokers, as the difference in TV between heavy smokers and non-smokers was reduced after iodization of salt (45).

#### Iodine status: studies from other Nordic countries

A cross-sectional study of Swedish national data on UIC of children aged 6–12 years indicated adequate iodine nutrition, and there were no gender or age differences in median UIC of the children (46). This study provides evidence that the voluntary addition of iodine to salt since 1,936 at a level of 40–70 mg/kg is sufficient to ensure adequate iodine nutrition in the Swedish population (46). Iodized table salt remains the main dietary source of iodine in the diet and among adults it is estimated to provide more than 50% of the iodine intake in Sweden (46). In another Swedish cross-sectional study among small groups of children, teenagers, and adults, the median UIC suggested adequate iodine nutrition (47).

A cross-sectional study including adolescent girls from Iceland found optimal iodine status; however, the result should be used with cation, as only 39% completed the study. Still the results are good estimates of the iodine nutrition of adolescent girls from Iceland (48).

Results from a representative study in Norway suggest that the dietary iodine intake is in the range considered to be sufficient among adults and children; however, it decreased among adolescents, especially among girls (49). Regular intake of milk, dairy products, and seafood are of importance to secure adequate iodine intake in Norway as the iodization of salt (only table salt) is very low (5 µg/g). This was clearly shown in the study including subjects with a variable intake of fish and dairy products, which indicated mild iodine deficiency among subjects having low intake of these two food groups (50).

## Discussion

Iodine deficiency remains a major threat to the health and development of populations around the world, and it is claimed that much of Europe is iodine deficient (51). The iodine status in all the Nordic countries is not well documented; however, based on UIC, the iodine nutrition status in Denmark, Iceland, Finland, and Sweden is sufficient and it is deficient in Norway according to WHO data (51).

The overall aim was to review recent scientific data on health effects of iodine status (as an indicator of iodine intake) in order to update current Nordic dietary reference values and to assess the scientific evidence and special relevance for the Nordic setting by increasing the RDI of iodine during pregnancy and lactation from what was presented in the 4th edition of NNR.

Grading of evidence is presented in Table 8. It should be emphasized that the grading of evidence is only based on studies from 2000–2010 and in some cases inclusion of earlier studies might have resulted in different grading. Evidence supporting that iodine supplementation during pregnancy is associated with maternal iodine status and thyroid function is suggestive (7, 9). One A study and one B study showed improved cognitive function of infants and children up to 18 months with potential indicators of improved iodine status of the mother (15, 16), while the evidence for improved cognitive function in older children is limited. It should be noted that no direct measurements of iodine intake where used in these studies (15, 16), and the conclusions are therefore based on the association between thyroid function (as an indicator of iodine status) and cognitive function of the offspring. The relevance of these studies to be used to set recommendations on iodine intake might therefore be questioned. Moderately to severely iodine-deficient children (6–13 years) will probably benefit from iodine supplementation or improved iodine status in order to improve cognitive function (23–25, 31), while only one study showed improved cognitive function with iodine supplementation in children from a mildly iodine-deficient area (23). No conclusions can be drawn related to other outcomes included in our search. A second literature search (using the same search string as previously) was conducted in March 2012, including studies published in the period October 2010 to February 2012. No additional studies were included in this review, as it would not modify the conclusions drawn from the studies included.

**Table 8:** Grading of evidence

	Grading of evidence*	Number of studies	References
Iodine supplementation during pregnancy is related to improved maternal iodine status and/or thyroid function.	Suggestive	Two B studies	Antonangeli et al. 2002 (7); Nøhr & Laurberg, 2000 (9)
Iodine supplementation during pregnancy is related to infant thyroid function.	No conclusion	One B study	Nøhr & Laurberg, 2000 (9)
Iodine supplementation of pregnant women with thyroid peroxidase antibodies (TPO-Ab) is not associated with adverse effects.			
No conclusion	One A study	Nøhr et al., 2000 (8)	
Improved prenatal iodine status is associated with improved cognitive function of infants and children up to 18 months.	Suggestive	One A study and two B studies	Oken et al. 2009 (16); Choudhury & Gorman 2003 (15)
Improved prenatal iodine status is associated with improved cognitive function in children above 2 years.	No conclusion	One A study	Oken et al. 2009 (16)
Iodine supplementation improves iodine status in mildly, moderately and severe iodine-deficient children (7–13 years).	Probable	Four B studies	Gordon et al., 2009 (23); Zimmermann et al., 2006 (24); van den Briel et al., 2000 (25); Zimmerman et al., 2009 (31).
Iodine supplementation or improved iodine status improves cognitive function in moderately to severe iodine-deficient children aged 6–13 years.	Probable	Three B studies	Gordon et al., 2009 (23); Zimmermann et al., 2006 (24); van den Briel et al., 2000 (25)
Iodine supplementation improves cognitive function in mildly iodine-deficient children aged 10–13 years	No conclusion	One B study	Gordon et al., 2009 (23)
Iodine supplementation is related to improved lipid profile in children	No conclusion	One B study	Zimmerman et al., 2009 (31)
Iodine status of adults is associated with features of the metabolic syndrome.	No conclusion	One B study	Ayturk et al., 2009 (32)
Iodine status is related to risk of prostate cancer.	No conclusion	One B study	Hoption Cann et al., 2007 (33)
Excessive intake of iodine (UIC 300–500 or >500 mcg/L) is associated with adverse effects in children.	No conclusion	One B study	Zimmermann et al., 2005 (34)

* Using the criteria for assigning grade of evidence presented in the WCRF cancer report, introduced in the SLR guide for the 5th edition of NNR.

Surprisingly, dietary data was only included in a very low number of studies. Furthermore, in many cases the exposure was thyroid function rather than estimate of iodine intake (i.e. UIC). Definitions of severe, moderate, and mild iodine deficiency also vary between studies. It is therefore challenging to use information from the studies included in this review in order to set dietary reference values.

## Conclusions

There are no new data supporting changes in dietary reference values for children or adults. Although the WHO/UNICEF/ICCIDD has increased the RDI for iodine from 200 to 250 µg/day in pregnancy and in lactating women (6), they emphasized the need for more data on the level of iodine intake that ensures maternal and newborn euthyroidism. The iodine requirement during pregnancy is increased because the mother synthesizes ∼50% more iodine-containing thyroid hormones to maintain maternal euthyroidism and to transfer thyroid hormones to the fetus and because the mother has increased renal losses of iodine (3). The rationale for increasing the dietary reference values for pregnant and lactating women in the 5th edition of NNR needs to be discussed in a broad perspective taking into account iodine status of pregnant women in the Nordic countries. Nordic studies retrieved have mainly described the thyroid function rather than the intake and sources of iodine in the diet. Further studies are required, especially among the most vulnerable groups, but also studies which assess possible adverse effects of high intake of iodine.

## Appendix 1

Search terms

**Date:** September 2010

**Database:** PubMed/Medline

(Humans[MeSH Terms]) OR human*[Title/Abstract]

Iodine[MeSH Terms]

Growth and development[Title/Abstract]) OR Thyroid gland[MeSH Terms]) OR Thyroid gland size[Title/Abstract]) OR Thyroid hormones[MeSH Terms]) OR Metabolism[Title/Abstract]) OR Hyperthyroidism[MeSH Terms]) OR Hypothyroidism[MeSH Terms]) OR Overweight[Title/Abstract]) OR Obesity[MeSH Terms]) OR Pregnancy[MeSH Terms]) OR pregnancy*[Title/Abstract]) OR Life style[Title/Abstract]) OR excessive[Title/Abstract]) OR insufficient[Title/Abstract]) OR iron[MeSH Terms]) OR selenium[MeSH Terms]) OR Urinary iodine concentration[Title/Abstract]) OR Iodine status[Title/Abstract]) OR Maternal Iodine intake[Title/Abstract]) OR Neonatal TSH[Title/Abstract]) OR cognition[Title/Abstract]) OR child development[MeSH Terms]) OR Child development[Title/Abstract]) OR infant development[Title/Abstract]) OR maternal iodine status[Title/Abstract]]

(1504 hits)

Search terms

**Date:** September 2010

**Database:** SveMed+Iodine[MeSH Terms] (12 hits)

## Appendix 2

**Table:**

Article	Reason for exclusion
(2003). “Iodine deficiency in Europe. National reports on iodine status in West-Central European countries. First symposium of ICCIDD West-Central Europe. Goteborg, Sweden, September 7, 2002.” *J Endocrinol Invest* **26**(9 Suppl): 1–62.	Overview.
Ainy, E., et al. (2007). “Assessment of intertrimester and seasonal variations of urinary iodine concentration during pregnancy in an iodine-replete area.” *Clin Endocrinol (Oxf)* **67**(4): 577–581.	Not relevant, only describing the status, no other outcome or food. From Iran.
Alikasifoglu, A., et al. (2002). “Serum insulin-like growth factor-I (IGF-I) and IGF-binding protein-3 levels in severe iodine deficiency.” *Turk J Pediatr* **44**(3): 215–218.	Cross sectional, the follow up is presented in Özon 2004.
Alvarez-Pedrerol, M., et al. (2009). “Organochlorine compounds, iodine intake, and thyroid hormone levels during pregnancy.” *Environ Sci Technol* **43**(20): 7909–7915.	Not relevant to the research questions.
Alvarez-Pedrerol, M., et al. (2010). “Iodine sources and iodine levels in pregnant women from an area without known iodine deficiency.” *Clin Endocrinol (Oxf)* **72**(1): 81–86.	Cross-sectional.
Aminorroaya, A., et al. (2004). “Iodine repletion, thyrotoxicosis and atrial fibrillation in Isfahan, Iran.” *Ann Saudi Med* **24**(1): 13–17.	From Iran, case report.
Andersen, S., et al. (2001). “Variations in urinary iodine excretion and thyroid function. A 1-year study in healthy men.” *Eur J Endocrinol* **144**(5): 461–465.	Methodology, includes 16 participants.
Andersson, M., et al. (2007). “Prevention and control of iodine deficiency in pregnant and lactating women and in children less than 2-years-old: conclusions and recommendations of the Technical Consultation.” *Public Health Nutr* **10**(12A): 1606–1611.	Review.
Andersson, M., et al. (2010). “Epidemiology of iodine deficiency: Salt iodisation and iodine status.” *Best Pract Res Clin Endocrinol Metab* **24**(1): 1–11.	Review.
Andersson, M., et al. (2005). “Current global iodine status and progress over the last decade towards the elimination of iodine deficiency.” *Bull World Health Organ* **83**(7): 518–525.	Review.
Andersson, M., et al. (2003). “The WHO Global Database on iodine deficiency disorders: the importance of monitoring iodine.” *Scandinavian Journal of Nutrition* **47**(4): 162–166.	Short communication.
Andersen, S., et al. (2002). “Iodine content of traditional Greenlandic food items and tap water in East and West Greenland.” Int J Circumpolar Health 61(4): 332–340.	Not related to the research question.
Angermayr, L., et al. (2004). “Iodine supplementation for preventing iodine deficiency disorders in children.” *Cochrane Database Syst Rev*(2): CD003819.	Cochrane review.
Aquaron, R., et al. (2002). “Bioavailability of seaweed iodine in human beings.” *Cell Mol Biol (Noisy-le-grand)* **48**(5): 563–569.	Not relevant for our research questions.
Ares, S., et al. (2008). “Iodine balance, iatrogenic excess, and thyroid dysfunction in premature newborns.” *Semin Perinatol* **32**(6): 407–412.	Report from seminars in perinatology.
Ares, S., et al. (2005). “Neonatal iodine deficiency: clinical aspects.” *J Pediatr Endocrinol Metab* **18 Suppl 1**: 1257–1264.	Review.
Arum, S. M., et al. (2009). “Excess iodine from an unexpected source.” *N Engl J Med* **360**(4): 424–426.	Letter to the editor, case report.
Azizi, F., et al. (2005). “Reappraisal of the risk of iodine-induced hyperthyroidism: an epidemiological population survey.” *J Endocrinol Invest* **28**(1): 23–29.	Observational, population study from Iran, not relevant.
Azizi, F., et al. (2009). “Breastfeeding and maternal and infant iodine nutrition.” *Clin Endocrinol (Oxf)* **70**(5): 803–809.	Review.
Bastemir, M., et al. (2006). “WITHDRAWN: High prevalence of thyroid dysfunction and autoimmune thyroiditis in adolescents after elimination of iodine deficiency in the Eastern Black Sea Region of Turkey.” *Thyroid* **16**(12): 1265–1271.	Withdrawn.
Becker, D. V., et al. (2006). “Iodine supplementation for pregnancy and lactation-United States and Canada: recommendations of the American Thyroid Association.” *Thyroid* **16**(10): 949–951.	Recommendation of the American Thyroid Association of iodine intake in US and Canada.
Benton, D. (2010). “The influence of dietary status on the cognitive performance of children.” *Mol Nutr Food Res* **54**(4): 457–470.	Review.
Berbel, P., et al. (2007). “Iodine supplementation during pregnancy: a public health challenge.” *Trends Endocrinol Metab* **18**(9): 338–343.	Opinion. Review.
Berkovski, V. (2002). “New iodine models family for simulation of short-term biokinetics processes, pregnancy and lactation.” *Food Nutr Bull* **23**(3 Suppl): 87–94.	Methodological aspect, outside our research questions.
Bernal, J. (2005). “Pathophysiology of thyroid hormone deficiency during fetal development.” *J Pediatr Endocrinol Metab* **18 Suppl 1**: 1253–1256.	Review.
Black, R. E., et al. (2008). “Maternal and child undernutrition: global and regional exposures and health consequences.” *Lancet* **371**(9608): 243–260.	Review.
Boas, M., et al. (2009). “Association of thyroid gland volume, serum insulin-like growth factor-I, and anthropometric variables in euthyroid prepubertal children.” *J Clin Endocrinol Metab* **94**(10): 4031–4035.	Cross-sectional with longitudinal data on growth. About thyroid function and not dietary iodine.
Bonar, B. D., et al. (2000). “Hypothyroidism and aging: the Rosses’ survey.” *Thyroid* **10**(9): 821–827.	Descriptive study from Ireland, nothing about iodine intake.
Borak, J. (2005). “Neonatal hypothyroidism due to maternal vegan diet.” *J Pediatr Endocrinol Metab* **18**(6): 621.	Letter to the editor.
Boric, M., et al. (2009). “Iodine supplementation in pregnancy.” *Acta Clin Croat* **48**(4): 469–473.	Review.
Bournaud, C., et al. (2003). “Iodine excess and thyroid autoimmunity.” *J Endocrinol Invest* **26**(2 Suppl): 49–56.	Review.
Brauer, V. F., et al. (2006). “Selenium and goiter prevalence in borderline iodine sufficiency.” *Eur J Endocrinol* **155**(6): 807–812.	Not relevant to the research question.
Braverman, L. E. (2001). “The physiology and pathophysiology of iodine and the thyroid.” *Thyroid* **11**(5): 405.	Guest editorial.
Brantsaeter, A. L., et al. (2009). “Evaluation of urinary iodine excretion as a biomarker for intake of milk and dairy products in pregnant women in the Norwegian Mother and Child Cohort Study (MoBa).” Eur J Clin Nutr 63(3): 347–354.	Not related to the research question.
Bruce, B., et al. (2003). “Isoflavone supplements do not affect thyroid function in iodine-replete postmenopausal women.” *J Med Food* **6**(4): 309–316.	Few participants, about thyroid function and nothing about iodine intake.
Bulow Pedersen, I., et al. (2006). “Increase in incidence of hyperthyroidism predominantly occurs in young people after iodine fortification of salt in Denmark.” *J Clin Endocrinol Metab* **91**(10): 3830–3834.	Salt fortification and hyperthyroidism. Cases of overt hyperthyroidism in two areas in DK as endpoints.
Burgi, H. (2010). “Iodine excess.” *Best Pract Res Clin Endocrinol Metab* **24**(1): 107–115.	Review.
Burns, R., et al. (2008). “Can neonatal TSH screening reflect trends in population iodine intake?” *Thyroid* **18**(8): 883–888.	Methodological aspect, outside our research questions.
Busnardo, B., et al. (2006). “Restricted intraindividual urinary iodine concentration variability in nonfasting subjects.” *Eur J Clin Nutr* **60**(3): 421–425.	Methodological aspect, outside our research questions.
Caldwell, K. L., et al. (2005). "Urinary iodine concentration: United States National Health And Nutrition Examination Survey 2001–2002." *Thyroid* **15**(7): 692–699.	UIC in US from NHANES. Descriptive study.
Camargo, R. Y., et al. (2008). “Thyroid and the environment: exposure to excessive nutritional iodine increases the prevalence of thyroid disorders in Sao Paulo, Brazil.” *Eur J Endocrinol* **159**(3): 293–299.	Only cross-sectional descriptive, without relating UIC to outcomes.
Cann, S. A., et al. (2000). “Hypothesis: iodine, selenium and the development of breast cancer.” *Cancer Causes Control* **11**(2): 121–127.	Review.
Carle, A., et al. (2006). “Epidemiology of subtypes of hypothyroidism in Denmark.” *Eur J Endocrinol* **154**(1): 21–28.	Describes incidences of subtypes of hypothyroidism before mandatory salt fortification.
Cerqueira, C., et al. (2009). "Association of iodine fortification with incident use of antithyroid medication–a Danish Nationwide Study." *J Clin Endocrinol Metab* **94**(7): 2400–2405.	Use of antithyroid medication as endpoint after mandatory fortification in Denmark.
Chanoine, J. P. (2003). “Selenium and thyroid function in infants, children and adolescents.” *Biofactors* **19**(3–4): 137–143.	Review.
Charnley, G. (2008). “Perchlorate: overview of risks and regulation.” *Food Chem Toxicol* **46**(7): 2307–2315.	Not relevant to the research questions. Also a review.
Clar, C., et al. (2002). “Iodized salt for iodine deficiency disorders. A systematic review.” *Endocrinol Metab Clin North Am* **31**(3): 681–698.	Not relevant to the research questions.
Dabbaghmanesh, M. H., et al. (2007). “Low serum selenium concentration as a possible factor for persistent goiter in Iranian school children.” *Biofactors* **29**(2–3): 77–82.	Cross-sectional study, no food, Iran with high goitre prevalence.
de Benoist, B., et al. (2008). “Iodine deficiency in 2007: global progress since 2003.” *Food Nutr Bull* **29**(3): 195–202.	Review.
de Escobar, G. M., et al. (2008). “The changing role of maternal thyroid hormone in fetal brain development.” *Semin Perinatol* **32**(6): 380–386.	Review. Seminars in perinatology.
de Escobar, G. M., et al. (2007). “Iodine deficiency and brain development in the first half of pregnancy.” *Public Health Nutr* **10**(12A): 1554–1570.	Review.
de Vijlder, J. J. (2003). “Primary congenital hypothyroidism: defects in iodine pathways.” *Eur J Endocrinol* **149**(4): 247–256.	Prize lecture.
Delange, F. (2000). “The role of iodine in brain development.” *Proc Nutr Soc* **59**(1): 75–79.	Review.
Delange, F. (2001). “Iodine deficiency as a cause of brain damage.” *Postgrad Med J* **77**(906): 217–220.	Editorial overview.
Delange, F. (2002). “Iodine deficiency in Europe and its consequences: an update.” *Eur J Nucl Med Mol Imaging* **29 Suppl 2**: S404–416.	Review.
Delange, F. (2005). “Epidemiology and impact of iodine deficiency in pediatrics.” *J Pediatr Endocrinol Metab* **18 Suppl 1**: 1245–1251.	Review.
Delange, F. (2007). “Iodine requirements during pregnancy, lactation and the neonatal period and indicators of optimal iodine nutrition.” *Public Health Nutr* **10**(12A): 1571–1580; discussion 1581–1573.	Review.
Delange, F., et al. (2002). “World status of monitoring iodine deficiency disorders control programs.” *Thyroid* **12**(10): 915–924.	Review.
Delange, F., et al. (2002). “Determining median urinary iodine concentration that indicates adequate iodine intake at population level.” *Bull World Health Organ* **80**(8): 633–636.	Descriptive study about UIC at population level from several countries in Europe.
Delange, F., et al. (2001). “Iodine deficiency in the world: where do we stand at the turn of the century?” *Thyroid* **11**(5): 437–447.	Review.
Delange, F., et al. (2000). “Iodine supplementation: benefits outweigh risks.” *Drug Saf* **22**(2): 89–95.	Current opinion. Review.
Delange, F., et al. (2000). “Silent iodine prophylaxis in Western Europe only partly corrects iodine deficiency; the case of Belgium.” *Eur J Endocrinol* **143**(2): 189–196.	The objective is to describe status in Belgium. We have similar stories from the Nordic countries
Derwahl, M., et al. (2000). “Multinodular goitre: ‘much more to it than simply iodine deficiency’.” *Baillieres Best Pract Res Clin Endocrinol Metab* **14**(4): 577–600.	Review.
Dorairajan, N., et al. (2002). “A descriptive study of papillary thyroid carcinoma in a teaching hospital in Chennai, India.” *Asian J Surg* **25**(4): 300–303.	Cancer patients, concluding that high intake is the main risk factor, but don't include food. Not relevant.
Dorea, J. G. (2002). “Iodine nutrition and breast feeding.” *J Trace Elem Med Biol* **16**(4): 207–220.	Review.
Dorey, C. M., et al. (2008). “Reference values for spot urinary iodine concentrations in iodine-sufficient newborns using a new pad collection method.” *Thyroid* **18**(3): 347–352.	Methodological aspect, outside our research questions
Dorr, M., et al. (2008). “The association of thyroid function with carotid artery plaque burden and strokes in a population-based sample from a previously iodine-deficient area.” *Eur J Endocrinol* **159**(2): 145–152.	Cross sectional study, says nothing about iodine intake.
Duarte, G. C., et al. (2009). “Excessive iodine intake and ultrasonographic thyroid abnormalities in schoolchildren.” *J Pediatr Endocrinol Metab* **22**(4): 327–334.	High intake not linked to any outcome in the paper, thyroid volume and UIC nothing about dietary intake.
Dunn, J. T. (2001). “Endemic goiter and cretinism: an update on iodine status.” *J Pediatr Endocrinol Metab* **14 Suppl 6**: 1469–1473.	Review.
Dunn, J. T. (2003). “Iodine should be routinely added to complementary foods.” *J Nutr* **133**(9): 3008S–3010S.	About fortification of foods.
Dunn, J. T., et al. (2001). “Damaged reproduction: the most important consequence of iodine deficiency.” *J Clin Endocrinol Metab* **86**(6): 2360–2363.	Commentary.
Dunn, J. T., et al. (2001). “Update on intrathyroidal iodine metabolism.” *Thyroid* **11**(5): 407–414.	Review.
Duntas, L. H. (2008). “Environmental factors and autoimmune thyroiditis.” *Nat Clin Pract Endocrinol Metab* **4**(8): 454–460.	Review
Elnour, A., et al. (2000). “Endemic goiter with iodine sufficiency: a possible role for the consumption of pearl millet in the etiology of endemic goiter.” *Am J Clin Nutr* **71**(1): 59–66.	Not relevant, Sudan, pearl millet, very iodine-deficient people.
Eltom, A., et al. (2000). “Thyroglobulin in serum as an indicator of iodine status during pregnancy.” *Scand J Clin Lab Invest* **60**(1): 1–7.	Methodological paper assessing status in the mother (Sudan and Swedish), no food or supplement use described in the paper, not relevant to research questions.
Eltom, A., et al. (2000). “Changes in iodine metabolism during late pregnancy and lactation: a longitudinal study among Sudanese women.” *Eur J Clin Nutr* **54**(5): 429–433.	Sudan, describing status in the mother, no food or supplement use described in the paper.
Eltom, A., et al. (2001). “Thyroid function in the newborn in relation to maternal thyroid status during labour in a mild iodine deficiency endemic area in Sudan.” *Clin Endocrinol (Oxf)* **55**(4): 485–490.	Not relevant to the research questions.
Fadeyev, V., et al. (2003). “Prevalence of thyroid disorders in pregnant women with mild iodine deficiency.” *Gynecol Endocrinol* **17**(5): 413–418.	Prevalence/cross-sectional, not Nordic and does not make any exact conclusion, none outcome in the infant.
Farahati, J., et al. (2006). “Gender-specific determinants of goiter.” *Biol Trace Elem Res* **113**(3): 223–230.	Only descriptive, nothing about iodine intake.
Fields, C., et al. (2005). “Iodine-deficient vegetarians: a hypothetical perchlorate-susceptible population?” *Regul Toxicol Pharmacol* **42**(1): 37–46.	Not relevant to the research question. Perclorate. Review of US vegetarian.
Galanti, M. R., et al. (2007). “Smoking and environmental iodine as risk factors for thyroiditis among parous women.” *Eur J Epidemiol* **22**(7): 467–472.	Smoking, risk of overt thyroiditis, nothing about iodine intake.
Gartner, R. (2009). “Thyroid diseases in pregnancy.” *Curr Opin Obstet Gynecol* **21**(6): 501–507.	Review.
Gatseva, P. D., et al. (2005). “Iodine status of children living in areas with high nitrate levels in water.” *Arch Environ Occup Health* **60**(6): 317–319.	Not relevant to our research questions – cross sectional study about nitrate intake in children in Bulgaria.
Giray, B., et al. (2001). “Status of selenium and antioxidant enzymes of goitrous children is lower than healthy controls and nongoitrous children with high iodine deficiency.” *Biol Trace Elem Res* **82**(1–3): 35–52.	Not relevant for our research questions, cross sectional study from Turkey.
Girelli, M. E., et al. (2004). “Milk represents an important source of iodine in schoolchildren of the Veneto region, Italy.” *J Endocrinol Invest* **27**(8): 709–713.	Descriptive, importance of milk in childhood in Italy.
Glinoer, D. (2001). “Pregnancy and iodine.” *Thyroid* **11**(5): 471–481.	Review.
Glinoer, D. (2003). “Feto-maternal repercussions of iodine deficiency during pregnancy. An update.” *Ann Endocrinol (Paris)* **64**(1): 37–44.	Review.
Glinoer, D. (2004). “The regulation of thyroid function during normal pregnancy: importance of the iodine nutrition status.” *Best Pract Res Clin Endocrinol Metab* **18**(2): 133–152.	Review
Glinoer, D. (2006). “Iodine nutrition requirements during pregnancy.” *Thyroid* **16**(10): 947–948.	Guest editorial.
Glinoer, D. (2007). “Clinical and biological consequences of iodine deficiency during pregnancy.” *Endocr Dev* **10**: 62–85.	Review.
Glinoer, D. (2007). “The importance of iodine nutrition during pregnancy.” *Public Health Nutr* **10**(12A): 1542–1546.	Review.
Glinoer, D., et al. (2009). “Gestational hypothyroxinemia and the beneficial effects of early dietary iodine fortification.” *Thyroid* **19**(5): 431–434.	Guest editorial.
Golkowski, F., et al. (2007). “Increased prevalence of hyperthyroidism as an early and transient side-effect of implementing iodine prophylaxis.” *Public Health Nutr* **10**(8): 799–802.	From Poland. Descriptive about hyperthyroidism. Similar effects reported from Denmark.
Grantham-McGregor, S. M., et al. (2000). “Nutritional deficiencies and later behavioural development.” *Proc Nutr Soc* **59**(1): 47–54.	Review.
Gregory, C. O., et al. (2009). “Use of supplements with and without iodine in women of childbearing age in the United States.” *Thyroid* **19**(9): 1019–1020.	Comment to editor.
Guan, H., et al. (2008). “Influence of iodine on the reference interval of TSH and the optimal interval of TSH: results of a follow-up study in areas with different iodine intakes.” *Clin Endocrinol (Oxf)* **69**(1): 136–141.	The aim is to determine a reference interval for TSH in a Chinese population.
Gunnarsdottir, I., et al. (2009). “Iodine intake and status in Iceland through a period of 60 years.” *Food & Nutrition Research* **53**(27 May): 1–4.	Review
Guo, T. W., et al. (2005). “Polymorphisms in the TSHR (thyrotropin receptor) gene on chromosome 14q31 are not associated with mental retardation in the iodine-deficient areas of China.” *Neurosci Lett* **382**(1–2): 179–184.	Not related to the research questions
Guo, T. W., et al. (2004). “Positive association of the DIO2 (deiodinase type 2) gene with mental retardation in the iodine-deficient areas of China.” *J Med Genet* **41**(8): 585–590.	Not related to the research questions
Hashemipour, M., et al. (2008). “Goiter persistence after iodine replenishment, the potential role of selenium deficiency in goitrous schoolchildren of Semirom, Iran.” *Exp Clin Endocrinol Diabetes* **116**(2): 75–79.	Cross sectional inconclusive from Iran.
Hays, M. T. (2001). “Estimation of total body iodine content in normal young men.” *Thyroid* **11**(7): 671–675.	Include only 6 subjects.
Hess, S. Y. (2010). “The impact of common micronutrient deficiencies on iodine and thyroid metabolism: the evidence from human studies.” *Best Pract Res Clin Endocrinol Metab* **24**(1): 117–132.	Review.
Hess, S. Y., et al. (2004). “The effect of micronutrient deficiencies on iodine nutrition and thyroid metabolism.” *Int J Vitam Nutr Res* **74**(2): 103–115.	Review.
Hess, S. Y., et al. (2001). “Monitoring the adequacy of salt iodization in Switzerland: a national study of school children and pregnant women.” *Eur J Clin Nutr* **55**(3): 162–166.	Only status, salt iodine increased on the population level, no information on individual intake.
Hetzel, B. S. (2000). “Iodine and neuropsychological development.” *J Nutr* **130**(2S Suppl): 493S–495S.	From a symposium on trace element and human health.
Hollowell, J. G., et al. (2002). “Serum TSH, T(4), and thyroid antibodies in the United States population (1988 to 1994): National Health and Nutrition Examination Survey (NHANES III).” *J Clin Endocrinol Metab* **87**(2): 489–499.	Thyroid levels in US population.
Hoogendoorn, E. H., et al. (2006). “Thyroid function and prevalence of anti-thyroperoxidase antibodies in a population with borderline sufficient iodine intake: influences of age and sex.” *Clin Chem* **52**(1): 104–111.	Not relevant to iodine intake (lack of information) thyroid function, prevalence.
Hoption Cann, S. A. (2006). “Hypothesis: dietary iodine intake in the etiology of cardiovascular disease.” *J Am Coll Nutr* **25**(1): 1–11.	Review.
Horton, S. (2006). “The economics of food fortification.” *J Nutr* **136**(4): 1068–1071.	Symposium. Review.
Huszno, B., et al. (2003). “Influence of iodine deficiency and iodine prophylaxis on thyroid cancer histotypes and incidence in endemic goiter area.” *J Endocrinol Invest* **26**(2 Suppl): 71–76.	Inconclusive, about thyroid cancer and radiation, intake based on several different studies in different age groups.
Ibrahim, M., et al. (2006). “Iodine supplementation for the prevention of mortality and adverse neurodevelopmental outcomes in preterm infants.” *Cochrane Database Syst Rev*(2): CD005253.	Cochrane review
Not related to our research question, supplementation in preterm infants (related to mortality).	
Kabelitz, M., et al. (2003). “The prevalence of anti-thyroid peroxidase antibodies and autoimmune thyroiditis in children and adolescents in an iodine replete area.” *Eur J Endocrinol* **148**(3): 301–307.	Cross sectional study.
Kaloumenou, I., et al. (2007). “Thyroid volume and echostructure in schoolchildren living in an iodine-replete area: relation to age, pubertal stage, and body mass index.” *Thyroid* **17**(9): 875–881.	Descriptive, cross-sectional study, not including any specific endpoint except function of the thyroid.
Kaloumenou, I., et al. (2008). “Thyroid autoimmunity in schoolchildren in an area with long-standing iodine sufficiency: correlation with gender, pubertal stage, and maternal thyroid autoimmunity.” *Thyroid* **18**(7): 747–754.	Cross sectional study
Karmisholt, J., et al. (2008). “Serum TSH and serum thyroid peroxidase antibody fluctuate in parallel and high urinary iodine excretion predicts subsequent thyroid failure in a 1-year study of patients with untreated subclinical hypothyroidism.” *Eur J Endocrinol* **158**(2): 209–215.	Descriptive study, include 21 sublinical hypothyroidism subjects
Knobel, M., et al. (2007). “Relevance of iodine intake as a reputed predisposing factor for thyroid cancer.” *Arq Bras Endocrinol Metabol* **51**(5): 701–712.	Review.
Knudsen, N., et al. (2001). "Serum Tg–a sensitive marker of thyroid abnormalities and iodine deficiency in epidemiological studies." *J Clin Endocrinol Metab* **86**(8): 3599–3603.	Methodological paper, outside research question.
Knudsen, N., et al. (2003). “Low socio-economic status and familial occurrence of goitre are associated with a high prevalence of goitre.” *Eur J Epidemiol* **18**(2): 175–181.	Descriptive study, socio-economic status and goitre.
	
Knudsen, N., et al. (2001). “Alcohol consumption is associated with reduced prevalence of goitre and solitary thyroid nodules.” *Clin Endocrinol (Oxf)* **55**(1): 41–46.	Descriptive study about alcohol consumption and goitre. Thyroid volume as endpoint measure.
Knudsen, N., et al. (2002). “High occurrence of thyroid multinodularity and low occurrence of subclinical hypothyroidism among tobacco smokers in a large population study.” *J Endocrinol* **175**(3): 571–576.	Smoking and thyroid function, includes nothing about iodine intake.
Knudsen, N., et al. (2002). “Low goitre prevalence among users of oral contraceptives in a population sample of 3712 women.” *Clin Endocrinol (Oxf)* **57**(1): 71–76.	Descriptive study about goitre and use of oral contraceptives.
Knudsen, N., et al. (2006). “Iodine and metabolic diseases. Consequences of iodine deficiency.” *Månedsskrift for praktisk laegegerning* **84**(12): 1317–1323.	Overview.
Knudsen, N., et al. (2002). “Parity is associated with increased thyroid volume solely among smokers in an area with moderate to mild iodine deficiency.” Eur J Endocrinol **146**(1): 39–43.	Not related to the research question.
Kunachowicz, H., et al. (2002). “Studies on iodine content in daily diets, particularly elderly people's diets.” *J Nutr Health Aging* **6**(2): 127–129.	Descriptive from Poland, not relevant.
Kung, A. W., et al. (2000). “Goitrogenesis during pregnancy and neonatal hypothyroxinaemia in a borderline iodine sufficient area.” *Clin Endocrinol (Oxf)* **53**(6): 725–731.	Not relevant.
Langer, P., et al. (2007). “Fish from industrially polluted freshwater as the main source of organochlorinated pollutants and increased frequency of thyroid disorders and dysglycemia.” *Chemosphere* **67**(9): S379–385.	Not relevant.
Langer, P., et al. (2007). “Thyroid ultrasound volume, structure and function after long-term high exposure of large population to polychlorinated biphenyls, pesticides and dioxin.” *Chemosphere* **69**(1): 118–127.	Almost the same as Langer et al. 2007 about fish from industrially polluted freshwater.
Lamberg, B. A. (2003). “[Iodine deficiency exists in many European countries but not in Finland].” *Duodecim* **119**(17): 1639–1642.	Review
Langer, P., et al. (2003). “Multimodal distribution versus logarithmic transformation of thyroid volumes in adolescents: detection of subgroup with subclinical thyroid disorders and its impact on the assessment of the upper limit of normal thyroid volumes.” *Endocr J* **50**(2): 117–125.	Methodological paper, outside research question.
Laurberg, P. (2005). “Global or Gaelic epidemic of hypothyroidism?” *Lancet* **365**(9461): 738–740.	Comment on paper.
Laurberg, P. (2009). “Thyroid function: Thyroid hormones, iodine and the brain-an important concern.” *Nat Rev Endocrinol* **5**(9): 475–476.	Short overview.
Laurberg, P., et al. (2007). “Evaluating iodine deficiency in pregnant women and young infants-complex physiology with a risk of misinterpretation.” *Public Health Nutr* **10**(12A): 1547–1552; discussion 1553.	Review methods for assessing iodine status.
Laurberg, P., et al. (2002). “Thiocyanate in food and iodine in milk: from domestic animal feeding to improved understanding of cretinism.” *Thyroid* **12**(10): 897–902.	Review.
Laurberg, P., et al. (2001). “Environmental iodine intake affects the type of nonmalignant thyroid disease.” *Thyroid* **11**(5): 457–469.	Review.
Laurberg, P., et al. (2010). “Iodine intake as a determinant of thyroid disorders in populations.” *Best Pract Res Clin Endocrinol Metab* **24**(1): 13–27.	Review, however included in the SLR as it is from the Nordic countries.
Laurberg, P., et al. (2006). “The Danish investigation on iodine intake and thyroid disease, DanThyr: status and perspectives.” *Eur J Endocrinol* **155**(2): 219–228.	Review.
Laurberg, P., et al. (2000). “Thyroid disorders in mild iodine deficiency.” *Thyroid* **10**(11): 951–963.	Review.
	
Lazarus, J. H., et al. (2004). “Prevalence of iodine deficiency worldwide.” *Lancet* **363**(9412): 901.	Correspondence.
Li, M., et al. (2010). “Neonatal TSH screening: is it a sensitive and reliable tool for monitoring iodine status in populations?” *Best Pract Res Clin Endocrinol Metab* **24**(1): 63–75.	Methodological paper about neonatal TSH screening, outside research question.
Li, Y., et al. (2008). “Antithyroperoxidase and antithyroglobulin antibodies in a five-year follow-up survey of populations with different iodine intakes.” *J Clin Endocrinol Metab* **93**(5): 1751–1757.	From China, cross sectional study, about thyroid function.
Mahomed, K., et al. (2000). “WITHDRAWN: Maternal iodine supplements in areas of deficiency.” *Cochrane Database Syst Rev*(2): CD000135.	Withdrawn.
Mahomed, K., et al. (2006). “WITHDRAWN: Maternal iodine supplements in areas of deficiency.” *Cochrane Database Syst Rev*(3): CD000135.	Withdrawn.
Manz, F., et al. (2000). “Iodine supply in children from different european areas: the Euro-growth study. Committee for the Study of Iodine Supply in European Children.” *J Pediatr Gastroenterol Nutr* **31 Suppl 1**: S72–75.	Cross sectional study with UIC as endpoint.
Mason, J. B., et al. (2002). “Iodine fortification is related to increased weight-for-age and birthweight in children in Asia.” *Food Nutr Bull* **23**(3): 292–308.	Inconclusive results (cross-sectional mainly) and not related to Nordic nutrition (conducted in Asia).
Melse-Boonstra, A., et al. (2010). “Iodine deficiency in pregnancy, infancy and childhood and its consequences for brain development.” *Best Pract Res Clin Endocrinol Metab* **24**(1): 29–38.	Review.
Mian, C., et al. (2009). “Iodine status in pregnancy: role of dietary habits and geographical origin.” *Clin Endocrinol (Oxf)* **70**(5): 776–780.	Review.
Milakovic, M., et al. (2006). “Effect of lifelong iodine supplementation on thyroid 131-I uptake: a decrease in uptake in euthyroid but not hyperthyroid individuals compared to observations 50 years ago.” *Eur J Clin Nutr* **60**(2): 210–213.	Descriptive study, 131-I uptake as endpoint.
Milerova, J., et al. (2006). “Actual levels of soy phytoestrogens in children correlate with thyroid laboratory parameters.” *Clin Chem Lab Med* **44**(2): 171–174.	Not relevant to the research questions.
Mirmiran, P., et al. (2002). “Three-year survey of effects of iodized oil injection in schoolchildren with iodine deficiency disorders.” *Exp Clin Endocrinol Diabetes* **110**(8): 393–397.	Not relevant for the Nordic countries, covered by the Cohrane review.
Mithen, R. (2007). “Effect of genotype on micronutrient absorption and metabolism: a review of iron, copper, iodine and selenium, and folates.” *Int J Vitam Nutr Res* **77**(3): 205–216.	Review.
Morreale de Escobar, G., et al. (2004). “Role of thyroid hormone during early brain development.” *Eur J Endocrinol* **151 Suppl 3**: U25–37.	Review
Mukhopadhyay, S., et al. (2005). “Evaluation of possible goitrogenic and anti-thyroidal effect of nitrate, a potential environmental pollutant.” *Indian J Physiol Pharmacol* **49**(3): 284–288.	Animal study about nitrate exposure.
Nishiyama, S., et al. (2004). “Transient hypothyroidism or persistent hyperthyrotropinemia in neonates born to mothers with excessive iodine intake.” *Thyroid* **14**(12): 1077–1083.	Iodine excess intake from seaweed as kombu.
Oberlin, O., et al. (2006). “Goitre and iodine deficiency in Afghanistan: a case-control study.” Br J Nutr **95**(1): 196–203.	Case-control, descriptive (goiter and thyroid function), aim of study was to identify whether the occurrence of goiter is a satisfactory marker of iodine deficiency.
Obregon, M. J., et al. (2005). “The effects of iodine deficiency on thyroid hormone deiodination.” *Thyroid* **15**(8): 917–929.	Review about changes caused by iodine deficiency in thyroid hormone metabolism.
Ohara, N., et al. (2004). “The role of thyroid hormone in trophoblast function, early pregnancy maintenance, and fetal neurodevelopment.” *J Obstet Gynaecol Can* **26**(11): 982–990.	Not relevant
Ovesen, L., et al. (2002). “The use of biomarkers in multicentric studies with particular consideration of iodine, sodium, iron, folate and vitamin D.” *Eur J Clin Nutr* **56 Suppl 2**: S12–17.	Methodological paper, outside research question.
Papanastasiou, L., et al. (2007). “Thyroid autoimmunity in the current iodine environment.” *Thyroid* **17**(8): 729–739.	Review.
Prakash, R. (2005). “High thyroid volume in children with excess dietary iodine intakes.” Am J Clin Nutr **82**(3): 708–709.	Letter to the editor
Patrick, L. (2008). “Iodine: deficiency and therapeutic considerations.” *Altern Med Rev* **13**(2): 116–127.	Review.
Pearce, E. N. (2009). “What do we know about iodine supplementation in pregnancy?” *J Clin Endocrinol Metab* **94**(9): 3188–3190.	Editorial comment.
Pearce, E. N., et al. (2002). “Effects of chronic iodine excess in a cohort of long-term American workers in West Africa.” *J Clin Endocrinol Metab* **87**(12): 5499–5502.	High intake due to high content of iodine in drinking water.
Pearce, E. N., et al. (2007). “Breast milk iodine and perchlorate concentrations in lactating Boston-area women.” *J Clin Endocrinol Metab* **92**(5): 1673–1677.	Not relevant.
Pedersen, I. B., et al. (2007). “An increased incidence of overt hypothyroidism after iodine fortification of salt in Denmark: a prospective population study.” *J Clin Endocrinol Metab* **92**(8): 3122–3127.	Cases of overt hypothyroidism in two areas in DK, salt fortification and cases of hypothyroidism.
Pemberton, H. N., et al. (2005). “Thyroid hormones and fetal brain development.” *Minerva Ginecol* **57**(4): 367–378.	Review.
Perez-Lopez, F. R. (2007). “Iodine and thyroid hormones during pregnancy and postpartum.” *Gynecol Endocrinol* **23**(7): 414–428.	Review.
Pizzulli, A., et al. (2000). “Selenium deficiency and hypothyroidism: a new etiology in the differential diagnosis of hypothyroidism in children.” *Biol Trace Elem Res* **77**(3): 199–208.	Case report.
Qian, M., et al. (2005). “The effects of iodine on intelligence in children: a meta-analysis of studies conducted in China.” *Asia Pac J Clin Nutr* **14**(1): 32–42.	Limited to studies from China, not relevant in the Nordic setting.
Radetti, G., et al. (2002). “Foetal and neonatal thyroid disorders.” *Minerva Pediatr* **54**(5): 383–400.	Review.
Rasmussen, L. B., et al. (2002). “Relations between various measures of iodine intake and thyroid volume, thyroid nodularity, and serum thyroglobulin.” *Am J Clin Nutr* **76**(5): 1069–1076.	Methodological issues. Not related to the research question.
Rayburn, W. F., et al. (2008). “Iodide concentrations in matched maternal serum, cord serum, and amniotic fluid from preterm and term human pregnancies.” *Reprod Toxicol* **25**(1): 129–132.	Short communication, lack of information, only iodine status of the mother, no endpoint in the fetus/infant.
Raymond, J., et al. (2010). “Fetal and neonatal thyroid function: review and summary of significant new findings.” *Curr Opin Endocrinol Diabetes Obes* **17**(1): 1–7.	Review.
Rebagliato, M., et al. (2010). “Iodine intake and maternal thyroid function during pregnancy.” *Epidemiology* **21**(1): 62–69.	Review
Remer, T., et al. (2006). “Longitudinal examination of 24-h urinary iodine excretion in schoolchildren as a sensitive, hydration status-independent research tool for studying iodine status.” *Am J Clin Nutr* **83**(3): 639–646.	Longitudinal descriptive study about the 24 h urinary iodine excretion in German children.
Restani, P., et al. (2008). “Analysis of food supplements containing iodine: a survey of Italian market.” *Clin Toxicol (Phila)* **46**(4): 282–286.	Supplement analysis of iodine in Italy.
Ristic-Medic, D., et al. (2009). “Methods of assessment of iodine status in humans: a systematic review.” *Am J Clin Nutr* **89**(6): 2052S–2069S.	Methodological paper, outside research question.
Robbins, J., et al. (2001). “Iodine nutrition and the risk from radioactive iodine: a workshop report in the Chernobyl long-term follow-up study.” *Thyroid* **11**(5): 487–491.	Workshop report. Review.
Rogahn, J., et al. (2000). “Randomised trial of iodine intake and thyroid status in preterm infants.” *Arch Dis Child Fetal Neonatal Ed* **83**(2): F86–90.	Preterm infants, not related to the research question (trial where diff amounts of iodine is given in preterm formula).
Rotondi, M., et al. (2000). “Parity as a thyroid size-determining factor in areas with moderate iodine deficiency.” *J Clin Endocrinol Metab* **85**(12): 4534–4537.	Not related to nutrition.
Sack, J. (2003). “Thyroid function in pregnancy-maternal-fetal relationship in health and disease.” *Pediatr Endocrinol Rev* **1 Suppl 2**: 170–176; discussion 176.	Review.
Savin, S., et al. (2003). “Thyroid hormone synthesis and storage in the thyroid gland of human neonates.” *J Pediatr Endocrinol Metab* **16**(4): 521–528.	No information on iodine intake or status. Very preterm infants and preterm infants (not healthy and died in the first month).
Serreau, R., et al. (2004). “Fetal thyroid goiter after massive iodine exposure.” *Prenat Diagn* **24**(9): 751–753.	Letter and a case report (short).
Sethi, V., et al. (2004). “Iodine deficiency and development of brain.” *Indian J Pediatr* **71**(4): 325–329.	Review.
Siklar, Z., et al. (2002). “Borderline congenital hypothyroidism in the neonatal period.” *J Pediatr Endocrinol Metab* **15**(6): 817–821.	Descriptive and not relevant to Nordic diet, children with jaundice.
Smyth, P. P. (2003). “The thyroid, iodine and breast cancer.” *Breast Cancer Res* **5**(5): 235–238.	Commentary about breast cancer and thyroid disease.
Smyth, P. P., et al. (2007). “Short-term changes in maternal and neonatal urinary iodine excretion.” *Thyroid* **17**(3): 219–222.	Not related to nutrition, small sample and short time.
Soldin, O. P., et al. (2003). “Urinary iodine percentile ranges in the United States.” *Clin Chim Acta* **328**(1–2): 185–190.	UIC values from US.
Soldin, O. P., et al. (2004). “Trimester-specific changes in maternal thyroid hormone, thyrotropin, and thyroglobulin concentrations during gestation: trends and associations across trimesters in iodine sufficiency.” *Thyroid* **14**(12): 1084–1090.	Not relevant to the research question, only describing the changes in hormones in the pregnant mother.
Soldin, O. P., et al. (2005). “Do thyroxine and thyroid-stimulating hormone levels reflect urinary iodine concentrations?” *Ther Drug Monit* **27**(2): 178–185.	Methodological aspect, outside our research questions.
Soriguer, F., et al. (2009). “Clinical dilemmas arising from the increased intake of iodine in the Spanish population and the recommendation for systematic prescription of potassium iodide in pregnant and lactating women (Consensus of the TDY Working Group of SEEN).” *J Endocrinol Invest* **32**(2): 184–191.	Overview.
Steinmaus, C., et al. (2007). "Impact of smoking and thiocyanate on perchlorate and thyroid hormone associations in the 2001–2002 national health and nutrition examination survey." *Environ Health Perspect* **115**(9): 1333–1338.	Not relevant to nutrition, mainly perchlorate.
Sullivan, K. M. (2007). “Iodine supplementation for pregnancy and lactation: United States and Canada: recommendations of the American Thyroid Association.” *Thyroid* **17**(5): 483–484.	Letter to the editor.
Takats, I. K., et al. (2000). “The blood spot thyrotropin method is not adequate to screen for hypothyroidism in the elderly living in abundant-iodine intake areas: comparison to sensitive thyrotropin measurements.” *Thyroid* **10**(1): 79–85.	Methodological aspect, outside our research questions.
Teas, J., et al. (2007). “Seaweed and soy: companion foods in Asian cuisine and their effects on thyroid function in American women.” *J Med Food* **10**(1): 90–100.	Few participant, semi relevant to research questions, about soy and seaweed.
Teng, W., et al. (2006). “Effect of iodine intake on thyroid diseases in China.” *N Engl J Med* **354**(26): 2783–2793.	From China, iodine supplementation and thyroid disease, does not include dietary intake data.
Teng, X., et al. (2008). “Safe range of iodine intake levels: a comparative study of thyroid diseases in three women population cohorts with slightly different iodine intake levels.” *Biol Trace Elem Res* **121**(1): 23–30.	Methodological aspect, outside our research questions.
Thomas Jde, V., et al. (2009). “Perinatal goiter with increased iodine uptake and hypothyroidism due to excess maternal iodine ingestion.” *Horm Res* **72**(6): 344–347.	Case report, include 8 cases from Brazil.
Thomson, C. D., et al. (2009). “Selenium and iodine supplementation: effect on thyroid function of older New Zealanders.” *Am J Clin Nutr* **90**(4): 1038–1046.	From New Zeeland.
Triggiani, V., et al. (2004). “Prospective study of post-partum thyroid immune dysfunctions in type 1 diabetic women and in a healthy control group living in a mild iodine deficient area.” *Immunopharmacol Immunotoxicol* **26**(2): 215–224.	Women diabetic, small number of subjects (*n*=28).
Untoro, J., et al. (2007). “Reaching optimal iodine nutrition in pregnant and lactating women and young children: programmatic recommendations.” *Public Health Nutr* **10**(12A): 1527–1529.	Editorial.
Untoro, J., et al. (2010). “The challenges of iodine supplementation: a public health programme perspective.” *Best Pract Res Clin Endocrinol Metab* **24**(1): 89–99.	Review.
Valentino, R., et al. (2004). “Screening a coastal population in Southern Italy: iodine deficiency and prevalence of goitre, nutritional aspects and cardiovascular risk factors.” *Nutr Metab Cardiovasc Dis* **14**(1): 15–19.	Cross sectional study from Italy.
Van Der Haar, F. (2007). “Goiter and other iodine deficiency disorders: a systematic review of epidemiological studies to deconstruct the complex web.” *Arch Med Res* **38**(5): 586–587; author reply 588–589.	Letter to the editor.
Vanderver, G. B., et al. (2007). “Cigarette smoking and iodine as hypothyroxinemic stressors in U.S. women of childbearing age: a NHANES III analysis.” *Thyroid* **17**(8): 741–746.	From USA, smoking and hypothyroxinemic.
Wang, H. Y., et al. (2000). “Apolipoprotein E is a genetic risk factor for fetal iodine deficiency disorder in China.” *Mol Psychiatry* **5**(4): 363–368.	From China, outside research questions.
Vejbjerg, P., et al. (2009). “Estimation of iodine intake from various urinary iodine measurements in population studies.” *Thyroid* **19**(11): 1281–1286.	Methodological paper, outside research question.
Vejbjerg, P., et al. (2009). “Thyroglobulin as a marker of iodine nutrition status in the general population.” *Eur J Endocrinol* 161(3): 475–481.	Methodological paper, outside research question.
Venturi, S., et al. (2000). “Role of iodine in evolution and carcinogenesis of thyroid, breast and stomach.” *Adv Clin Path* **4**(1): 11–17.	Review.
Venturi, S., et al. (2009). “Iodine in evolution of salivary glands and in oral health.” *Nutr Health* **20**(2): 119–134.	Methodological aspect, outside our research questions.
Verger, P., et al. (2001). “Iodine kinetics and effectiveness of stable iodine prophylaxis after intake of radioactive iodine: a review.” *Thyroid* **11**(4): 353–360.	Review.
Verheesen, R. H., et al. (2008). “Iodine deficiency, more than cretinism and goiter.” *Med Hypotheses* **71**(5): 645–648.	Review.
Vermiglio, F., et al. (2004). “Attention deficit and hyperactivity disorders in the offspring of mothers exposed to mild-moderate iodine deficiency: a possible novel iodine deficiency disorder in developed countries.” *J Clin Endocrinol Metab* **89**(12): 6054–6060.	Only 16 subjects.
Vestergaard, P., et al. (2002). “Smoking as a risk factor for Graves’ disease, toxic nodular goiter, and autoimmune hypothyroidism.” *Thyroid* **12**(1): 69–75.	Risk factors of Graves disease, says nothing about iodine intake.
Williams, G. R. (2008). “Neurodevelopmental and neurophysiological actions of thyroid hormone.” *J Neuroendocrinol* **20**(6): 784–794.	Review.
Vitti, P., et al. (2001). “Iodine deficiency disorders in Europe.” *Public Health Nutr* **4**(2B): 529–535.	Review
Wu, T., et al. (2002). “Iodised salt for preventing iodine deficiency disorders.” *Cochrane Database Syst Rev*(3): CD003204.	Review.
Zagrodzki, P., et al. (2000). “The role of selenium in iodine metabolism in children with goiter.” *Environ Health Perspect* **108**(1): 67–71.	Cross sectional study
from Poland.	
Zeisel, S. H. (2009). “Is maternal diet supplementation beneficial? Optimal development of infant depends on mother's diet.” *Am J Clin Nutr* **89**(2): 685S–687S.	Review.
Zhao, J., et al. (2000). “Endemic goiter associated with high iodine intake.” *Am J Public Health* **90**(10): 1633–1635.	Do not assess any health effects of high intake of iodine due to high levels in water.
Zimmermann, M., et al. (2000). “Low dose oral iodized oil for control of iodine deficiency in children.” *Br J Nutr* **84**(2): 139–141.	Intervention among very IDD children from Cote d'Ivoire – not relevant to the Nordic countries.
Zimmermann, M., et al. (2004). “Iodine supplementation of pregnant women in Europe: a review and recommendations.” *Eur J Clin Nutr* **58**(7): 979–984.	Review.
Zimmermann, M. B. (2002). “Iron status influences the efficacy of iodine prophylaxis in goitrous children in Cote d'Ivoire.” *Int J Vitam Nutr Res* **72**(1): 19–25.	Interventions focusing on improvement of IDD in areas of endemic goiter in children are not relevant to the Nordic setting. We don't have as severe problems.
Zimmermann, M. B. (2004). “Assessing iodine status and monitoring progress of iodized salt programs.” *J Nutr* **134**(7): 1673–1677.	Methodological aspect, outside our research questions.
Zimmermann, M. B. (2007). “The adverse effects of mild-to-moderate iodine deficiency during pregnancy and childhood: a review.” *Thyroid* **17**(9): 829–835.	Review.
Zimmermann, M. B. (2007). “The impact of iodised salt or iodine supplements on iodine status during pregnancy, lactation and infancy.” *Public Health Nutr* **10**(12A): 1584–1595.	Review.
Zimmermann, M. B. (2008). “Iodine requirements and the risks and benefits of correcting iodine deficiency in populations.” *J Trace Elem Med Biol* **22**(2): 81–92.	Review.
Zimmermann, M. B. (2009). “Iodine deficiency.” *Endocr Rev* **30**(4): 376–408.	Review.
Zimmermann, M. B. (2009). “Iodine deficiency in pregnancy and the effects of maternal iodine supplementation on the offspring: a review.” *Am J Clin Nutr* **89**(2): 668S–672S.	Review.
Zimmermann, M. B. (2010). “Symposium on ‘Geographical and geological influences on nutrition’: Iodine deficiency in industrialised countries.” *Proc Nutr Soc* **69**(1): 133–143.	Symposium review.
Zimmermann, M. B., et al. (2000). “Effect of oral iodized oil on thyroid size and thyroid hormone metabolism in children with concurrent selenium and iodine deficiency.” *Eur J Clin Nutr* **54**(3): 209–213.	Not relevant to Scandinavia, very deficient area where iodized oil is tested.
Zimmermann, M. B., et al. (2006). “Assessment of iodine status using dried blood spot thyroglobulin: development of reference material and establishment of an international reference range in iodine-sufficient children.” *J Clin Endocrinol Metab* **91**(12): 4881–4887.	Methodological aspect, outside our research questions.
Zimmermann, M. B., et al. (2008). “Iodine-deficiency disorders.” *Lancet* **372**(9645): 1251–1262.	Seminar and review.
Zimmermann, M. B., et al. (2002). “The impact of iron and selenium deficiencies on iodine and thyroid metabolism: biochemistry and relevance to public health.” *Thyroid* **12**(10): 867–878.	Review.
Zimmermann, M. B., et al. (2001). “Toward a consensus on reference values for thyroid volume in iodine-replete schoolchildren: results of a workshop on inter-observer and inter-equipment variation in sonographic measurement of thyroid volume.” *Eur J Endocrinol* **144**(3): 213–220.	Methodological aspect, outside our research questions.
Zimmermann, M. B., et al. (2003). “Introduction of iodized salt to severely iodine-deficient children does not provoke thyroid autoimmunity: a 1-year prospective trial in northern Morocco.” *Thyroid* **13**(2): 199–203.	Not relevant to the Nordic countries. Morocco.
Zimmermann, M. B., et al. (2004). “Rapid relapse of thyroid dysfunction and goiter in school-age children after discontinuation of salt iodization.” *Am J Clin Nutr* **79**(4): 642–645.	Including children with severe IDD, discontinuation of a iodized salt program and the consequences.
Zimmermann, M. B., et al. (2002). “Addition of microencapsulated iron to iodized salt improves the efficacy of iodine in goitrous, iron-deficient children: a randomized, double-blind, controlled trial.” *Eur J Endocrinol* **147**(6): 747–753.	Not relevant to the research question (Nordic countries), only supplementation and results on function.
Zois, C., et al. (2003). “High prevalence of autoimmune thyroiditis in schoolchildren after elimination of iodine deficiency in northwestern Greece.” *Thyroid* **13**(5): 485–489.	Supplementation in very IDD area. Not related to NNR

## Appendix 3

**Table:** (studies presented in summary table 1). Iodine status and iodine supplementation in pregnancy; pregnancy outcome and thyroid function in the mother and offspring.

Reference, details, first author year, country	Study design	Population, subject	Outcome measures	Intervention/exposure	Time between baseline exposure and outcome assessment	Dietary assessment method	No. of subjects analyzed	Intervention	Follow-up period, drop-out rate	Results	Confounders adjusted for	Study quality and relevance, Comments A-C
Antonangeli et al., 2002, Italy (7)	Clinical trial	pregnant women (*n*=86), 20–38 years, enrolled from 10th to the 16th week of gestation. Women with clinical and laboratory evidence of hyperthyroidism, hypothyroidism, thyroid autoimmunity (thyroid autoantibodies >1:400) or thyroid volume greater than 20 mL were excluded. After recruitment 7 women withdrew their consent and 12 dropped out of the study (eight because of serious gestational events). After randomization the UIE was 91 mcg/g creatinine in group A and 65.5 mcg/g creatinine in group B.	UIE (casual urinary samples), TV, FT4, FT3, TSH, Tg.	Group A received on table iodide 200 per day (200mcg/d) and group B received 1/2 tablet iodide 100 (50 mcg/day).	Subjects assessed at 18th-26th week, 29th-33rd week, 3rd and 6th month after delivery.	No assessment of dietary intake.	*n*=67 (A *n*=32 and B *n*=35).	200 mcg iodine vs. 50mcg iodine.	Follow-up period from the first trimester throughout pregnancy to 6 months after delivery (approx. 12–14 months). Drop-out 22.1%.	Six months after delivery UIC was 230 mg/g creatinine in group A and 128 mg/g creatinine in group B. No difference in TV, Thyroid function or clinical events found between groups.	None reported.	B No information how women were randomized into groups. Observed difference in UIE after randomization, but no statistical test presented if this difference is statistically significant. Neither information on lactation nor iodine intake from other sources reported.
Nøhr & Laurberg, 2000, Denmark (9)	Cohort study	Healthy pregnant women with no previous history of thyroid disease, from 5 different regions of Denmark (*n*=152). Women with regular daily intake of multivitamin and mineral tablet containing iodine (150 mcg) during pregnancy (+I group, *n*=50) and women with no artificial iodine supplementation (no I group, *n*=96) continued this study, whereas women who had a history of intermittent iodine supplementation (*n*=6) where excluded. Median UI in the +I group was 60 mcg/L and 34.5 mcg/L in the no I group.	Maternal and neonatal thyroid function. UI measured in a spot sample on day 5 after delivery.	The participants were instructed to continue their previous vitamin and mineral supplementation during the puerperal period. 150mcg/day or no artificial iodine. The women had been recommended to take vitamin and mineral supplementation as part of normal pregnancy care.	Mother and infant at term and infant on day 5.	Not reported.	*n*=146. Blood samples from mothers shortly after admission for labor *n*=144, mixed cord blood samples *n*=139.			Mothers in the +I group had lower TSH (mU/L), higher free T4 (nmol/L) and lower Tg (mg/L) than the no I group (median (25–75%); 2.06 (1.49–2.47) vs. 2.23 (1.65–3.08), 8.4 (7.5–9.7) vs. 7.9 (7.0–8.8) and 14.7 (7.1–25.2) vs. 25.8 (16.4–53.4), respectively, *p*<0.05. The neonates showed a pattern different from the mothers. The +I group of neonates had higher TSH than the no I group; 9.00 (6.18–14.81) vs. 7.07 (4.72–11.58), while T4 was higher and Tg was lower in the neonatal +I group than in the no I group, similar to that in the mothers.	Age, parity, gestational length or birth weight of the neonates were not different between groups.	B The study suggest that iodine supplementation of the mother will, in general, not improve fetal thyroid function in areas such as Denmark with mild iodine deficiency. A slightly inhibitory effect may be expected, which is probably not of clinical significance.
Nøhr et al., 2000, Denmark (8)	RCT, double blind trial	Women with thyroid peroxidase antibodies (TPO-Ab) *n*=117 (prevalence 9.1%) from the healthy pregnant Danish women cohort (age 18–35 years, *n*=1284), screened at week 11 (median). 72 TPO-Ab women agreed to participate (61.5% of the eligible population). Randomised and stratified according to TPO-Ab level to three groups.	Postpartum thyroid dysfunction (PPTD) defined as abnormal TSH in the postpartum period (subclinical hypothyroidism if only TSH was abnormal and clinical hypothyroidism if TSH and thyroid hormones were abnormal).	150 mcg iodine supplement or no iodine. Group +/+ (*n*=22/22) pregnancy and postpartum, Group +/−(24/20 in pregnancy only and Group -/- (26/24) received no supplements.	Thyroid function evaluated at 11 w, 35 w, gestation and 3, 5, 7, and 9 months postpartum.	Compliance was evaluated by 24-h urinary iodine measurements at time of inclusion, 35 w of pregnancy and 7 months postpartum.	*n*=66		Follow-up from gestational week 11 throughout pregnancy and to 9 months postpartum. Drop-out 8%.	No statistical significant difference in the frequency of PPTD in the three groups, with no significant increase in the prevalence, severity, or duration of PPTD when 150 mcg iodine were given to TPO-Ab positive women during pregnancy only or during pregnancy and the post-partum period.	Smoking, group, age and parity.	A Unlikely that supplementation of 150mcg/day will have adverse effects in TPO-Ab women living in an area with mild to moderate ID.

## Appendix 4

**Table:** (studies presented in summary table 2). Prenatal iodine status and cognitive function in the offspring.

Reference, details, first author year, country	Study design	Population, subject	Outcome measures	Intervention/exposure	Time between baseline exposure and outcome assessment	No. of subjects analyzed	Follow-up period, drop-out rate	Results	Confounders adjusted for	Study quality and relevance, Comments A-C
Choudhury & Gorman 2003. China (15)	Cohort study	Infants (*n*=284) from a non-endemic region of Northern China. No information on how they were selected to the study. Stratification of infants into iodine deficiency groups (ID) by cord blood TSH concentration (group 1 (control) <5 mU/L, group 2: 10.0–19.9 mU/L, group 3: 20.0–29.9 mU/L, group 4 ≥30 mU/L). Gender distribution in groups approximately equal.	Infant information processing (FTII) at 7 months (*n*=275). Infant cognitive and motor development (BSID-II) at 13 months (*n*=135). The BSID-II was subdivided into mental development index (MDI) and psychomotor development index (PDI)	Prenatal iodine deficiency (cord blood TSH concentration); control group <5 mU/L, group 2: 10.0–19.9 mU/L, group 3: 20.0–29.9 mU/L and group 4 ≥30 mU/L.	7 and 13 months	*n*=275 at 7 months and *n*=135 at 13 months	FTII at 7 months available for 96% of the original group. BSID-II at 13 months available for 49% of the original group.	Infants in the highest TSH cord blood concentration groups (3 and 4), had lower novelty preference (57.7±5.6 and 57.5±3.1, respectively) (suggesting less efficient information processing) than infants in the non-elevated and the mildly elevated groups (1 and 2, 59.6±3.0 and 58.9±4.3, respectively, *p*<0.05). The three elevated TSH groups (2,3 and 4) had significantly lower MDI scores than the non-elevated control group (98.2±8.3, 98.7±9.3 and 93.5±11.1 vs. 102.5±8.2 respectively, *p*<0.05). No difference in PDI between groups.	Maternal education, place of residence (rural vs. urban) and maternal occupation.	B No information on iodine nutrition (neither urine iodine nor iodine intake). The overall novelty preference score and MDI score was well within the expected range in all groups.
Oken et al. 2009. USA (16)	Cohort study	Children, 6 months and 3 years (50.4% male) of mothers who enrolled in the Project Viva cohort between 1999 and 2002. Women attending their initial prenatal visit (*n*=2128) at one of eight urban and suburban obstetrical offices in a multi-specialty group practice in Massachusetts. Eligibility criteria included fluency in English, gestational age less than 22 wks, singleton pregnancy, and plans to remain in the study area. Of 2128 women in the Project Viva who delivered a live infant, 988 had info on first trimester diet and infant cognitive testing at 6 months, and were thus eligible for inclusion in the present study. Maternal T4 (mcg/dL) 9.98 ±1.95 (*n*=496).	Cognitive testing using the visual recognition memory (VRM) paradigm at 6 months. Cognition assessed using two tests at 3 years: the Peabody Picture Vocabulary Test (PPVT) and Wide Range Assessment of Visual Motor Ability WRAVMA)	New born T4 (thyroxine) levels and maternal thyroid function (plasma TSH, total T4 and TPO antibody levels)	6 months and 3 years	*n*=500 (missing data for some measurements).	6mo and 3 years. Out of 988 eligible, 500 gave consent.	Higher newborn T4 was associated with slightly lower scores on the VRM test at 6 months (-0.5;95%CI -0.9, -0.2). Newborn T4 levels were not associated with scores on either the PPVT or WRAVMA at age 3 years. No evidence that impaired maternal thyroid function was associated with lower child cognitive test scores.	Maternal age, race/ethnicity, education, post-partum depression, mode of delivery, smoking, first trimester thyroid function, fish intake, intake of iodine-containing vitamins, thyroid medication use during pregnancy, diagnosed thyroid disease did not change the effect estimates.	A No information on iodine nutrition (neither urine iodine nor iodine intake). Low number of women with abnormal thyroid function.

## Appendix 5

**Table:** (studies presented in summary table 3). Iodine supplementation or improved iodine status in childhood and cognitive function.

Reference, details, first author year, country	Study design	Population, subject	Outcome measures	Intervention/exposure	Time between baseline exposure and outcome assessment	Dietary assessment method	No. of subjects analyzed	Intervention	Follow-up period, drop-out rate	Results	Confounders adjusted for	Study quality and relevance, Comments A-C
Gordon et al., 2009. New Zeeland (Dunedin) (23)	RCT, double-blind.	Children, 10–13 years, no known history of thyroid conditions, not taking I supplement. 162 children recruited from schools, 22 from advertisement, all together 184. 55% boys. Baseline UIC 63 mcg/L, Thyroglobulin 16.4 mcg/L, Total Thyroxine 104±28.1 nmol/L.	Cognitive performance. Wechsler Intelligence Scale for Children. Subtests: Picture concepts, matrix reasoning, symbol search, letter-number sequencing.	150 mcg I tablet vs. Placebo.	28 wks	FFQ, foods considered as main sources of I. Caregiver completed a FFQ about intake of dairy products, milk, red meat, poultry, fish, shellfish, pulses and legumes, fruit, eggs, and iodized salt.	*n*=166. I group *n*=84 and placebo *n*= 82.	Children were provided with 4-wk supplements in 28-day compliance packaging blister packs and an information sheet how to take their supplements. Every 4 wks a new pack of supplements was posted. Return envelope included to collect previous months supplements. If a pack was not returned, the compliance was assumed to be zero for that month. Movie vouchers, small stationary items, or shopping vouchers were sent out during the study to aid with compliance.	11 drop-out in I group, 7 in placebo. Total drop-out 11%.	After 28 wk: I group UIC 145 mcg/L, Thyroglobulin 8.5 mcg/L. Placebo group UIC 81 mcg/L, Thyroglobulin 11.6 mcg/L. 2 of 4 cognitive subtest significantly improved in the I group. Perceptual reasoning in mildly ID children were improved in I group. Picture concept associated with 0.81 age-standardized point improvement in iodine relative to placebo (*p*=0.023), and 0.63 points in matrix reasoning (*p*=0.040).	Sex, method of recruitment, cohort, ethnicity and household income.	B Results are important and relevant in a Nordic perspective since the study is among children in mildly iodine-deficient area. Suggest that mild ID could prevent children from attaining their full intellectual potential.
Zimmermann et al., 2006. Albania (24)	RCT, double-blind	All children 10–12 years at 7 primary schools in villages in the Korce/Pogradec district of southeastern Albania were invited, *n*=310 (166 boys and 144 girls).	Morning spot urine, TSH, total thyroxine (TT4) and thyroid gland volume. 7 cognitive and motor skills tests (measures of information processing, working memory, visual problem solving, visual search and fine motor skills).	400 mg iodine as oral iodized oil vs. placebo.	24 wk	None, only UIC.	*n*=159 in I group and *n*= 151 in placebo group.	After baseline testing the children were randomly assigned to receive 400 mcg oral I as iodized poppy seed oil (Lipiodol) or a sunflower oil (placebo). The capsules were swallowed with water under direct supervision.	6 children moved and did not complete the cognitive retesting (4 in I group, 2 in placebo group) (4%). Thyroid function tests was not measured at follow up in 12% of the children because they refused blood sampling.	I group: UIC increased from 42 (0–186) to 172 (18–724) µg/L, Thyroid volume reduced from 5.9 (2.6–12.5) to 5.0 (2.4–9.7) mL, TSH unchanged 0.8 (0.3–2.5) and 0.7 (2.4–2.6), TT4 increased from 76 ±17 to 106±18 nmol/L. Placebo group: UIC unchanged 44 (0–215) and 49 (3–221), thyroid volume unchanged 6.2 (2.1–16.8) and 6.3 (2.6–16.0) mL, TSH unchanged 0.9 (0.4–2.6) 0.8 (0.2–7.7), TT4 unchanged 75 ±17 and 81 ±19 nmol/L. I group signifies improved performance on 4 of 7 tests (mean adjusted treatment effect (95% CI)): Rapid target marking 2.8 (1.6–4.0), symbol search 2.8 (1.9–3.6), rapid object naming 4.5 (2.3–6.6), and Raven's Coloured Progressive Matrices 4.7 (3.8–5.8).	Baseline difference between groups, sex, and school.	B Study from an iodine-deficient area in Albania. Might not be relevant for the Nordic countries.
van den Briel et al., 2000. West Africa (25)	RCT, double blind. Data treated as cohort study.	Children 6–12 years from four primary schools *n*=211 (approx. 85% boys).	Height, weight, blood (TSH, serum ferritin, Tg, free T4), urine. Mental test battery: closure, concentration, exclusion, fluency, mazes, hand movements, colored progressive matrices. Two psychomotor tests – pegboard and ball throwing.	Iodine supplement (1 mL iodized oil 540 g I/L) or placebo.	Baseline measurements in Oct and Nov 1995. repeated in Oct and Nov 1996.	None, only UIC.	Improved group (*n*=128), unchanged group (*n*=68).	As the population began to have access to iodized salt during the intervention period, the study population was split post hoc on the basis of UIC into group with improved iodine status and a group with unchanged iodine status (i.e. status changed from severe iodine deficiency to moderate, from severe to normal-mild, or from moderate to normal-mild).	13 children left school or moved and 2 children could not be located during urine collection. Drop-out 7%.	Children with increased UIC from baseline to endpoint (improved iodine status) had significantly greater increase in performance on the combination of mental tests than did the group with no change in UIC (0.12±0.06, *p*=01044).	Both groups consisted of supplemented and non-supplemented children, proportions not different. Also comparable in HB concentration, anthropometric and socioeconomic indexes and initial scores on the mental tests.	B Study includes schoolchildren in Benin and reflects not Nordic countries. The results suggest a “catch up” effects in terms of mental performance after iodine supplementation.

## Appendix 6

**Table:** (studies presented in summary table 4). Iodine status and health outcomes in adults and elderly.

Reference, details, first author year, country	Study design	Population, subject	Outcome measures	Intervention/exposure	Time between baseline exposure and outcome assessment	No. of subjects analyzed	Follow-up period, drop-out rate	Results	Confounders adjusted for	Study quality and relevance, Comments A-C
Ayturk et al., 2009. Turkey (32)	Case–control study.	New patients (18–74 years) with metabolic syndrome (*n*=278) living in a mild-to moderate iodine deficiency area, who attended for regular follow-up. 261 euthyroid control subjects without known thyroid disease recruited from patients admitting to family outpatient clinic. Matched according to age, gender, and smoking.	TSH, thyroid volume and nodule prevalence.			*n*=539; *n*=278 in the MetS group (33.1% male) and *n*=261 in the control group (30.7% male)		TSH was significantly correlated with the presence of MetS. In a multiple linear regression analysis, independent predictors of thyroid volume (mL) were (B, 95% CI); waist circumference (cm) 0.335 (0.089–0.161), triglycerides (mg/dL) 0.136 (0.003–0.016), and insulin resistance 0.143 (0.512–2.731).	BMI, smoking, fat-mass	B No information on iodine nutrition (neither urine iodine nor iodine intake). Patients with MetS have signifies increased thyroid volume and nodule prevalence. Insulin resistance is suggested as an independent risk factor for nodule formation in an iodine-deficient environment.
Hoption Cann et al., 2007. USA (33)	Cohort study.	NHEFS, 25–74 years, *n*=5811 males. Excluded due to lack of urinary iodine at baseline (*n*=1577), leaving 4234 men for analysis. Mean age at examination 52.7 years, 16.5% non-white. Tertiles of Iodine/Creatinine categories (<201 *n*=1452, 201–345 *n*=1554, >345 *n*=1228, referred to as low, moderate and high levels).	Prostate cancer incidence.	Iodine status (UIC and UI/Cr ratio). Spot samples.	Baseline in 1971 and 1975, Follow up in 1982–1984, 1986, 1987 and 1992).	*n*=4234 subjects, *n*=197 cases.	7–21 years Drop-out 10%.	Moderate I/Cr associated with a borderline increase risk of prostate cancer relative to low levels (HR=1.33 (95% CI 1.00–1.78). NS in the multivariate model (HR=1.31 (0.98–1.75)). High levels was associated with a reduced risk of prostate cancer, HR= 0.71 (0.51–0.99) but NS in the multivariate model (HR=0.75 (0.53–1.05)). Reported history of thyroid disease was associated with greater than twofold increased risk, HR=2.34 (1.24–4.43), remained significant after adjustment (HR=2.16 (1.13–4.14))	Age, race, marital status, family income, alcohol consumption at baseline, region	B The role of iodine remains speculative, a role of thyroid disease and/or factors contributing to thyroid disease as a risk factor for prostate carcinogenesis warrants additional investigation.

## Appendix 7

**Table:** (studies presented in summary table 5). Excessive intake of iodine.

Reference, details, first author year, country	Study design	Population, subject	Outcome measures	Intervention/exposure	Time between baseline exposure and outcome assessment	Dietary assessment method	No. of subjects analyzed	Intervention	Follow-up period, drop-out rate	Results	Confounders adjusted for	Study quality and relevance, Comments A-C
Zimmermannet al., 2005. Multiethnic (North and South America, Central Europe, Eastern Mediterranean, Africa and the Western Pacific) (34)	Cross-sectional.	6–12 year children primary schools whose pupils were of middle-to-low socioeconomic status.	Thyroid volume (by ultrasound measurement).	Iodine intake assessed by UIC in spot urine samples.		None, only UI.	*n*=3319. (*n*=534 from Switzerland, *n*=526 from Bahrain, *n*=591 from South Africa, *n*=524 from Peru, *n*=562 from Chelsea MA, *n*=302 from central Japan, *n*=280 from coastal Japan).			31% of children had UIC> 300 mcg/L and 11% > 500 mcg/L. UIC of 300–500 mcg/L not associated with increased Tvol. Tvol started to increase at a UIC ≈ 500 mcg/L.	Age, sex and body surface area (BSA).	B The authors don‘t rule out adverse effects of UIC in the range of 300–500 mcg/day not detected in this study.
